# Multi‐Isotopic Reconstruction of Prehistoric Diet and Trophic Organization in Cis‐Baikal Using Zinc and Amino Acid Nitrogen Isotopes

**DOI:** 10.1002/ajpa.70396

**Published:** 2026-11-09

**Authors:** Gwen Le Bras, Erin Jessup, Christelle Lagane, David Point, Rick J. Schulting, Victoria van der Haas, Ian Scharlotta, Stéphanie Mandrou, Olga I. Goriunova, Vladimir I. Bazaliiskii, Andrzej W. Weber, Klervia Jaouen

**Affiliations:** ^1^ Université de Toulouse, CNRS/IRD, Géosciences Environnement Toulouse Toulouse France; ^2^ Department of Anthropology University of Alberta Edmonton Alberta Canada; ^3^ School of Archaeology University of Oxford Oxford UK; ^4^ Center for Archaeological Studies Texas State University San Marcos Texas USA; ^5^ Research Centre “Baikal Region” Irkutsk State University Irkutsk Russia

**Keywords:** amino acid nitrogen isotopes, bronze age, dental enamel, freshwater ecosystems, hunter‐gatherers, Neolithic, non‐traditional stable isotopes, trophic level, zinc isotopes

## Abstract

**Objectives:**

Reconstructing dietary practices in prehistoric hunter‐gatherer populations requires disentangling complex ecological, cultural, and chronological variables. In the Cis‐Baikal region of Siberia, subsistence systems relied on combinations of freshwater and terrestrial resources, including fish, Baikal seals, and terrestrial game. This study integrates zinc isotope ratios (δ^66^Zn) from mineralized tissues with compound‐specific nitrogen isotope analysis of amino acids (CSIA‐AA) to evaluate the potential of δ^66^Zn to document trophic structure and dietary resource use in prehistoric human populations, with faunal data providing the ecological context across three microregions: Angara, Southwest Baikal, and the Little Sea.

**Materials and Methods:**

We analyzed δ^66^Zn values measured in dental enamel, dentin, and bone, together with CSIA‐AA nitrogen isotope data, from humans and associated fauna from three Cis‐Baikal microregions. Fish were classified into dietary clusters based on ecological feeding strategies to assess relationships between δ^66^Zn, diet, and trophic position.

**Results:**

Among human populations, zinc isotope ratios provide a sensitive proxy for identifying seal consumption within mixed subsistence systems. Phenylalanine nitrogen isotope values (δ^15^N_Phe_) discriminate between terrestrial and aquatic protein baselines. In freshwater fishes, δ^66^Zn values vary primarily according to dietary ecology rather than trophic level, indicating that dietary Zn isotope composition exerts a stronger control on tissue δ^66^Zn than trophic position.

**Discussion:**

The combined use of δ^66^Zn and CSIA‐AA refines dietary interpretations in complex terrestrial–freshwater ecosystems by disentangling baselines, trophic structure, and specific resource use. These findings demonstrate the utility of combining δ^66^Zn and CSIA‐AA to improve reconstructions of subsistence strategies in the studied prehistoric populations.

## 1 Introduction

Aquatic resources have played and continue to play an essential role in the diets of hunter‐gatherers worldwide. In coastal areas, their use has been well‐documented through mollusk accumulation and shell middens dating back to the earliest traces of human occupation (Marean 2014; Bailey and Milner 2002; Erlandson 2001). In freshwater ecosystems, however, documenting ancient aquatic resource use poses substantial challenges. Fish bones are less likely to be preserved, freshwater mollusks and mammals are less abundant, and traditional dietary proxies, such as bulk δ^13^C and δ^15^N analyses, may fail to distinguish between freshwater and terrestrial food sources due to overlapping isotopic ratios (Schoeninger et al. 1983; Post 2002; Vander Zanden et al. 1999; Bocherens et al. 2016). Recent advances in compound‐specific isotope analysis of amino acids (CSIA‐AA) have substantially improved reconstructions of trophic organization and freshwater resource consumption (Corr et al. 2005; Naito et al. 2010; Ohkouchi et al. 2017; Steffan et al. 2013), by disentangling baseline isotopic signals—with essential amino acids—from trophic level effects—with non‐essential amino acids (Chikaraishi et al. 2009; Larsen et al. 2013; McMahon et al. 2016; Steffan et al. 2013). However, the reliance of CSIA‐AA on preserved collagen limits its application in archeological contexts where collagen preservation is poor—especially in tropical or arid environments and in increasingly older samples (Ambrose 1991; Klinken 1999; Krigbaum 2005).

To address this limitation, several complementary isotopic approaches based on mineralized tissues have also been proposed to investigate dietary behavior when collagen is not preserved (Chu et al. 2006; Guiserix et al. 2022). Among these, zinc (Zn) isotopes have emerged as a promising alternative because they can be measured in dental enamel, a highly mineralized tissue that is resistant to diagenesis and preserves dietary information over much longer timescales (up to several million years) and under conditions where collagen is no longer preserved (Bourgon 2022; McCormack, Griffiths, et al. 2022). In addition, Zn isotope ratios (δ^66^Zn) can vary systematically with trophic levels due to isotopic fractionation during digestion and absorption or due to isotopic differences between tissues within the same organism. In vertebrates, a trophic level spacing of ~0.3‰–0.7‰ has been documented, supporting the use of δ^66^Zn as a dietary marker (Bourgon et al. 2020; Jaouen, Beasley, et al. 2016; Jaouen, Szpak, and Richards 2016; Jaouen et al. 2022). Although Zn isotopes have demonstrated considerable potential as dietary proxies in terrestrial and marine vertebrates, their application to freshwater ecosystems remains insufficiently evaluated. Freshwater food webs are characterized by complex and overlapping trophic pathways, and previous studies have reported weak or inconsistent δ^66^Zn‐based trophic structuring at the ecosystem scale (Nitzsche et al. 2020). Most freshwater applications also lack integrated prey–consumer datasets, limiting evaluation of whether tissue‐specific Δ^66^Zn offsets documented in terrestrial vertebrates are transferable to complex freshwater systems or to non‐mammalian taxa (e.g., crocodilians; Jaouen, Beasley, et al. 2016).

The Baikal Archaeology Project (BAP) has investigated prehistoric hunter‐gatherer populations (Neolithic and Bronze Age) of the Cis‐Baikal region since the 1990s (Katzenberg and Weber 1999; Lam 1994; Link and Weber 2001; Weber et al. 2002). The Cis‐Baikal region, encompassing the northwestern shores of Lake Baikal and its hinterland (Figure 1), is characterized by pronounced ecological diversity, including taiga, steppe, riverine and lacustrine environments, which supported a wide range of terrestrial and aquatic resources (Galazii 1993; Tarasov et al. 2007; Kobe et al. 2022). BAP research has documented mixed subsistence systems combining aquatic resources (fish and Baikal seals, locally known as nerpa) with terrestrial large game, with marked variation across microregions and periods, while terrestrial hunting remained critical (Weber 2020). Isotopic and zooarcheological evidence further indicate differences between Early Neolithic Kitoi and Early Bronze Age Glazkovo cultural patterns, particularly in the relative importance of lacustrine resources, mobility and social differentiation (Katzenberg et al. 2011; Waters Rist et al. 2021; Weber et al. 2002, 1998). This well‐documented framework provides an ideal testbed to evaluate the performance of δ^66^Zn alongside δ^15^N‐CSIA‐AA and to refine their combined application to complex ecological or archeological contexts.

**FIGURE 1:**
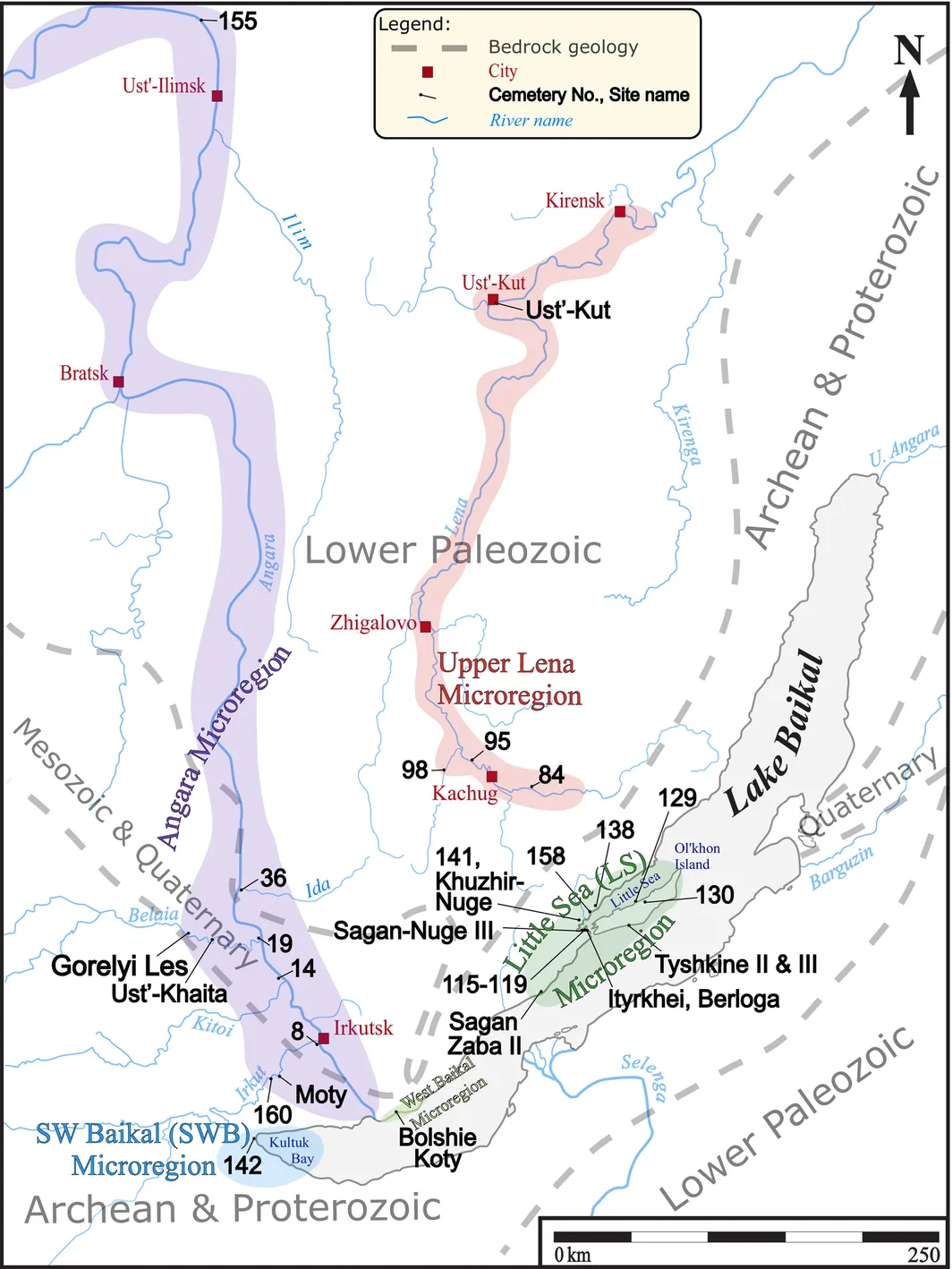
Map of the Cis‐Baikal region, showing the location of the Angara, Southwest Baikal (SW Baikal), and Little Sea microregions. Archeological sites are indicated by their cemetery numbers and names (see Table 1 for correspondence). Major bedrock geological boundaries are shown to provide geological context. After Bronk Ramsey et al. (2014) and Scharlotta and Weber (2012).

In addition to diet, δ^66^Zn values may also be influenced by geological and ecological baseline variations. Differences in bedrock lithology (Galazii 1993; Huh et al. 1994; Delvaux et al. 1997; Moynier et al. 2017) and associated soil Zn isotope compositions, as well as variation in primary producers and herbivore feeding ecologies (Viers et al. 2007; Pederzani et al. 2024; Martin and Jaouen 2024; Méjean et al. 2026), may contribute to baseline isotopic heterogeneity. This must be considered when interpreting trophic signals across the Angara, Southwest Baikal, and Little Sea microregions (Figure 1).

This study evaluates whether δ^66^Zn can distinguish terrestrial from aquatic resource consumption in prehistoric Cis‐Baikal populations and refine interpretations of Kitoi and Glazkovo subsistence strategies. δ^66^Zn values are combined with CSIA of amino acids (notably *δ* ^15^N_Phe_ and δ^15^N_Glx_) and other mobility or dietary proxies (newly generated and previously published bulk δ^13^C, δ^15^N, and Sr isotope ratios) to improve dietary reconstructions and trophic relationships. We analyze three cemetery populations representing distinct mortuary traditions and ecological settings (Figure 1; cemeteries 8, 142, and 141): Lokomotiv (Kitoi, Angara), Shamanka II (Kitoi, Southwest Baikal), and Khuzhir‐Nuge XIV (Glazkovo, Little Sea) (Haverkort et al. 2008; Katzenberg and Weber 1999; Weber et al. 2016a, 2016b, 2025; Weber 2020). These patterns are interpreted within mixed hunter‐gatherer economies that relied on terrestrial large game for dietary and non‐dietary needs, alongside differential use of seasonally available seal resources (Weber et al. 1993, 1998; Nomokonova et al. 2015). Human isotopic data are compared with associated faunal baselines, including modern fish where archeological preservation is limited.

## 2 Materials and Methods

### 2.1 Archeological Context

The study examines Early Neolithic Kitoi (7560–6660 cal. BP) and Early Bronze Age Glazkovo (4970–3470 cal. BP) hunter‐gatherers from the Cis‐Baikal region from three archeological microregions defined by the BAP: Angara, Southwest Baikal (SWB), and the Little Sea (LS, Figure 1). These geographically distinct zones encompass different aquatic environments and associated fisheries, including the Angara River and its tributaries, Kultuk Bay in Southwest Baikal, and the shores, coves, and open waters of the Little Sea. Their boundaries represent geographical and archeological subdivisions rather than exclusive ecological zones.

The Kitoi pattern is documented in the Angara and Southwest Baikal microregions, whereas the Little Sea assemblages considered here belong to the later Glazkovo pattern. All populations were exclusively hunter‐gatherers, without domesticates other than the dog (Weber 1995, 2020; Weber et al. 2025; Weber et al. 2021).

Previous isotopic research (δ^13^C, δ^15^N, ^87^Sr/^86^Sr) has documented marked dietary and mobility patterns. All individuals included in the present study have previously published bulk C, N, and Sr isotope data generated within the BAP, providing the archeological and isotopic framework used here to interpret the newly generated δ^66^Zn data. At Lokomotiv (Kitoi, Angara, Figure 1 and Table 1), individuals relied extensively on local fish, within mixed subsistence systems (Weber et al. 2021; Weber et al. 2002). At Shamanka II (Kitoi, SW Baikal), dietary groups 1, 2, and 5 show increasing reliance over time on lacustrine resources, including fish and possibly some seals (Weber et al. 2021; Weber et al. 2025). At Khuzhir‐Nuge XIV (Glazkovo, Little Sea), individuals were divided into two dietary groups: one combining game, fish, and seals (GFS) and another relying mainly on game and fish (GF) (Weber and Goriunova 2013; Weber and Bettinger 2010). Strontium isotope data suggested that most GF individuals originated outside the Little Sea microregion, with their isotope signatures being consistent with origins in either the Angara or Upper Lena valley (Scharlotta et al. 2018; Weber and Goriunova 2013; Weber et al. 2011).

**TABLE 1:** Summary of the human populations (A) and faunal reference (B) dataset analyzed in this study.

A. Human archeological populations						
Cemetery	Microregion	Archeological period	Date	Geology	*n* analyzed	No. Figure 1
Lokomotiv	Angara	Kitoi (EN)	7560–6660 cal. BP (Weber et al. 2021)	Limestones and dolomites	16	8
Shamanka II	SW Baikal	Kitoi (EN)	7560–7000 cal. BP (Weber et al. 2025)	Metamorphic and igneous rocks	15	142
Khuzhir‐Nuge XIV	Little Sea	Glazkovo (EBA)	4970–3470 cal. BP (Weber et al. 2021)	Paleozoic sedimentary rocks + Quaternary alluvial deposits	19	141

B. Faunal reference dataset by microregion					
Microregion	List of sites	Site No./name (Figure 1)	Chronological attribution	Species	Geology
Angara	Kitoi	14	Early Neolithic + Bronze Age + Modern	*Abramis brama* (*n* = 3) *Acipenser* sp. (*n* = 2) *Brachymystax lenok* (*n* = 3) *Capreolus pygargus* (*n* = 3) *Cervus elaphus* (*n* = 9) *Coregonus autumnalis* (*n* = 3) *Coregonus lavaretus* (*n* = 2) *Equus* sp. (*n* = 2) *Esox lucius* (*n* = 2) *Gobio gobio* (*n* = 3) *Hucho taimen* (*n* = 3) *Leuciscus leuciscus* (*n* = 3) *Lota lota* (*n* = 3) *Marmota sibirica* (*n* = 1) *Perca fluviatilis* (*n* = 3) *Sus scrofa* (*n* = 1) *Thymallus arcticus* (*n* = 3)	Limestones and dolomites
	Lokomotiv	8			
	Gorelyi Les	Gorelyi Les			
	Ostrov Listvenichnyi	155			
	Ust’‐Belaia	19			
	Ust’‐Ida I	36			
	Ust’‐Khaita	Ust’‐Khaita			
	Moty	Moty			
	Moty‐Novaia Shamanka	160			
SW Baikal	Kultuk Bay	Kultuk Bay	Early Neo‐lithic + Bronze Age + Modern	*Comephorus dybowskii* (*n* = 3) *Cottocomephorus grewingkii* (*n* = 3) *Esox lucius* (*n* = 2) *Leocottus kesslerii* (*n* = 3) *Marmota sibirica* (*n* = 2) *Perca fluviatilis* (*n* = 5) *Rutilus rutilus* (*n* = 3) *Ursus arctos* (*n* = 3)	Metamorphic and igneous rocks
	Shamanka II	142			
W Baikal	Bolshie Koty	Bolshie Koty	Modern	*Leuciscus idus* (*n* = 2) *Thymallus baicalensis* sp. (*n* = 4)	Metamorphic and igneous rocks
Lake Baikal	Lake Baikal	Lake Baikal	Modern	*Esox lucius* (*n* = 1) *Leuciscus idus* (*n* = 1) *Lota lota* (*n* = 1) *Thymallus baicalensis* (*n* = 2)	Metamorphic and igneous rocks
Little Sea	Berloga	Berloga	Late Mesolithic + Early Bronze Age + Modern	*Acipenser* sp. (*n* = 1) *Artiodactyla* sp. (*n* = 1) *Bos* sp. (*n* = 4) *Canidae* (*n* = 4) *Canis lupus familiaris* (*n* = 2) *Canis* sp. (*n* = 3) *Capreolus pygargus* (*n* = 1) *Cervus elaphus* (*n* = 4) *Coregonus baicalensis* (*n* = 2) *Coregonus migratorius* (*n* = 1) *Esox lucius* (*n* = 2) *Lota lota* (*n* = 1) *Pusa sibirica* (*n* = 28) *Rutilus rutilus* (*n* = 2) *Sus scrofa* (*n* = 1) *Thymallus baicalensis brevipinnis* (*n* = 3) *Ursus arctos* (*n* = 1)	Metamorphic and igneous rocks + Paleozoic sedimentary rocks
	Ithyrkhei	Ithyrkhei			
	Kharansa I	130			
	Khuzhir‐Nuge XIV	141			
	Khuzhir‐Nuge	Khuzhir‐Nuge			
	Kurma XI	138			
	Little Sea	Little Sea			
	Mys Uiuga	158			
	Sagan Nuge III	Sagan Nuge III			
	Sagan‐Zaba II	Sagan‐Zaba II			
	Shamanskii Mys	129			
	Tyshkine II and III	Tyshkine II and III			
	Ulan‐Khada	115–119			
Upper Lena	Upper Lena River	Upper Lena River	Modern	*Hucho taimen* (*n* = 1)	Paleozoic sedimentary rocks + Quaternary alluvial deposits
Upper Lena N	Ust’‐Kut	Ust’‐Kut	Modern	*Esox lucius* (*n* = 1)	
Upper Lena S	Obkhoi	98	Late Mesolithic + Neolithic	*Canis* sp. (*n* = 1) *Cervus elaphus* (*n* = 2) *Sus scrofa* (*n* = 1)	
	Makrushino	84			
	Nikolskii Grot	95			

*Note:* Detailed site‐by‐site information, including localities, geological context, taxa, tissue type, chronology, and analytical availability, is provided in Tables S2 and S3.

Abbreviations: EBA, early bronze age; EN, early neolithic.

Further archeological and chronological details are provided in the Supporting Information.

### 2.2 Material

The sampling strategy was designed to investigate human dietary variability across three well‐documented archeological microregions rather than to exhaustively characterize individual sites. Human populations were selected because they represent contrasting cultural, chronological, ecological, and previously reconstructed dietary contexts within the BAP (Figure 1). Faunal specimens were then assembled at the microregional scale to establish local isotopic baselines and evaluate environmental and trophic variability. Consequently, the faunal reference dataset integrates specimens from multiple archeological sites within each microregion, an approach reflecting their shared archeological and environmental context. Modern specimens were included only to complement archeological assemblages when particular taxa were unavailable or poorly represented in the archeological record due to preservation biases, particularly for fish remains (Table 1; see Supporting Information, Section 1.2). A total of 201 specimens (50 human and 151 faunal specimens) provided by the Baikal Archaeology Project (BAP) were analyzed at Géosciences Environnement Toulouse. Human samples derive from three Cis‐Baikal cemeteries: Lokomotiv (*n* = 16, Angara), Shamanka II (*n* = 15, SW Baikal), and Khuzhir‐Nuge XIV (*n* = 19, Little Sea; Figure 1, Table 1 and Table S2).

Zinc isotope analyses (δ^66^Zn) were conducted on 193 specimens, including bone, enamel, dentin, and whole fish, while single amino acid nitrogen isotope analyses (δ^15^N_AA_) were conducted on a subset of 63 samples (23 humans and 40 fauna, Table 1, Table S2). Not all specimens were analyzed for every isotopic proxy as both materials (collagen or dental enamel) were not always available. The type of tissue analyzed depended on specimen availability and preservation. The analytical coverage of each specimen is detailed in Table S3. Whenever possible, homologous tissues were analyzed within taxonomic groups. When this was not feasible, previously published tissue‐specific δ^66^Zn offsets were used to estimate equivalent bone δ^66^Zn values, thereby allowing inter‐specimen and inter‐taxon comparisons (see Section 2.3.2). Whole fish were analyzed for species with poorly mineralized skeletons because their bones are difficult to isolate and because whole‐body measurements better represent the material effectively consumed by piscivorous predators such as *Pusa sibirica* . Previously published sequential bulk δ^13^C and δ^15^N analyses had been performed on the same human teeth included in this study (Scharlotta et al. 2018; Haas 2023). The human dentin collagen sections used for δ^15^N‐CSIA‐AA analyses were selected from these previously generated sequential samples to correspond to the enamel formation periods analyzed for δ^66^Zn and to post‐weaning plateau sections of the sequential δ^15^N_bulk_ profiles, ensuring temporal comparability between the two proxies. Previously published bulk isotope data (δ^13^C, δ^15^N, and ^87^Sr/^86^Sr) were integrated to expand the comparative framework (Bronk Ramsey et al. 2014; Nomokonova et al. 2013; Schulting et al. 2014, 2015; Weber et al. 2002, 2011).

### 2.3 Methods

#### 2.3.1 Collagen Extraction and Bulk Carbon and Nitrogen Isotope Analysis

Collagen was extracted from bone and dentin samples following the protocol described by Fuller et al. (2014), involving demineralization in 0.5 M HCl, gelatinization at 70°C under weakly acidic conditions (pH~3), filtration and lyophilization. Bulk carbon and nitrogen isotope ratios were measured by EA‐IRMS at Iso‐Analytical Ltd. (UK) and calibrated against international reference materials traceable to VPDB (carbon) and AIR (nitrogen). Additional analytical details are provided in the Supporting Information (Section 1.3.1).

#### 2.3.2 Zinc Purification and Isotope Analyses

Samples were mechanically cleaned to remove surface contamination and prepared in a metal‐free clean laboratory using ultrapure reagents. Zn was extracted following established protocols (Moynier et al. 2006; Jaouen, Beasley, et al. 2016), with adaptations for fish samples based on McCormack, Bourgon, et al. (2022) allowing recovery of the mineral fraction during collagen extraction. Following digestion in 1 M HCl double distilled ultrapure acid (upa), all samples were evaporated and redissolved in 1.5 M HBr upa. Zinc was purified by ion exchange chromatography.

Zinc isotope ratios were determined using a Thermo Fisher Neptune Plus MC‐ICP‐MS (Observatoire Midi‐Pyrénées, Toulouse), following the protocol and cup configuration described by Toutain et al. (2008). The setup involved Cu‐doping for mass bias correction and sample‐standard bracketing to the in‐house AA‐MPI Zn standard. δ^66^Zn values are reported relative to the JMC‐Lyon standard. Analytical quality was monitored using blanks and reference materials (SRM‐1400). Mass‐dependent fractionation was monitored by also measuring δ^67^Zn and δ^68^Zn values (Figure S2A; *R* ^2^ = 0.98, *p* < 0.001; Albarède 2004). Each sample was analyzed in duplicate when possible.

When non‐bone tissues were analyzed, published taxon‐specific tissue offsets were applied to facilitate comparisons between individuals analyzed on different mineralized tissues. To allow inter‐tissue comparisons, δ^66^Zn values were converted to “bone‐equivalent” values using published offsets (variability reported as 1 SD): $\Delta^{66}{\mathrm{Zn}}_{\text{bone}-\text{enamel}}=+0.15‰$ for humans and seals (published offsets: +0.15‰ ± 0.16‰ and +0.15‰ ± 0.05‰ respectively, Jaouen et al. 2017; Fuller et al. 2022), $\Delta^{66}{\mathrm{Zn}}_{\text{bone}-\text{enamel}}\approx +0.20‰$ for terrestrial fauna (±0.18‰, Jaouen, Beasley, et al. 2016), and $\Delta^{66}{\mathrm{Zn}}_{\text{bone}-\text{whole fish}}=+0.45‰$ for whole fish samples (calculated from published inter‐tissue offsets from McCormack, Karnes, et al. 2023; McCormack et al. 2024). Dentin values were considered equivalent to bone (Bourgon et al. 2024; McCormack et al. 2024; McCormack, Griffiths, et al. 2022). Raw tissue‐specific and corrected δ^66^Zn data are available in Table S3.

#### 2.3.3 Strontium Isotope Complementary Analyses

To evaluate potential geological or baseline influences on δ^66^Zn values, we performed ^87^Sr/^86^Sr measurements for a subset of 48 samples. These analyses targeted the same mineralized structure and life stage as the Zn measurements and were compared with published Sr isotope data from the same individuals.

Samples were prepared following the protocol fully described in Britton (2010) and analyzed using a Thermo Fisher Triton Plus TIMS (Observatoire Midi‐Pyrénées, Toulouse). Analytical accuracy was monitored using SRM 1486 and isotope ratios were corrected relative to the accepted value of SRM 987 (see Table S5).

#### 2.3.4 Nitrogen Isotope Analyses of Single Amino Acids

Hydrolyzed collagen samples were derivatized by esterification of carboxyl groups with acidified 2‐propanol and acetylation of amino groups, following the protocol of Corr et al. (2007) as detailed in Ma et al. (2021). Single amino acid δ^15^N values were measured by GC‐IRMS at Géosciences Environnement Toulouse using a Thermo Fisher MAT 253 coupled to a Trace 1310 via ConFlo IV interface. Nitrogen isotope measurements are reported relative to atmospheric nitrogen (AIR). An internal standard of L‐Norleucine, international reference materials (USGS65, USGS74), and in‐house standards were included to monitor analytical accuracy. Additional procedural and instrumental details are provided in the Supporting Information (Section 1.3.3).

#### 2.3.5 Trophic Position Estimation

Trophic positions (TP) were estimated using both bulk collagen δ^15^N values and CSIA‐AA values (δ^15^N_Glx_ and δ^15^N_Phe_). They were compared to theoretical trophic positions (TP_theo_), which were retrieved for fish from the FishBase (Froese and Pauly 2025). For aquatic organisms, we applied the formula proposed by (Yoshii et al. 1999) for the Baikal pelagic food web:

$$\delta^{15}N_{\text{bulk}}=3.3\cdot \left({\mathrm{TP}}_{\text{bulk}}-1\right)+3.8$$

Rearranged, trophic position is estimated as:

$${\mathrm{TP}}_{\text{bulk}}=\frac{\left(\delta^{15}N_{\text{bulk}}-3.8\right)}{3.3}+1$$

This equation is particularly suited to Baikal fish due to the regional calibration of baseline δ^15^N values.

For all taxa with available CSIA‐AA data, TP_aq_ was also estimated using the difference in δ^15^N between glutamic acid and phenylalanine:

$${\mathrm{TP}}_{\mathrm{AA}}=1+\frac{\left(\delta^{15}N_{\mathrm{Glx}}-\delta^{15}N_{\mathrm{Phe}}-\beta \right)}{\mathrm{TDF}}$$

Hereafter, TP estimates calculated using the aquatic *β* value are referred to as TP_aq_, whereas those calculated using the terrestrial *β* value are referred to as TP_terr_. Both correspond to TP_AA_ estimates that differ only in the *β* parameter used. Following Chikaraishi et al. (2010), Ohkouchi et al. (2017) and Ramirez et al. (2021), we used a trophic discrimination factor (TDF) of 7.6‰ and initially set *β* = 3.4‰ for aquatic producers. Recent evidence suggests that the Glx–Phe TDF may be lower in carnivores than the conventional value used here (Krajcarz et al. 2025); therefore, TP_AA_ estimates for seals should be interpreted cautiously. For terrestrial herbivores, we calibrated the *β* parameter used in TP estimation by computing the mean offset between δ^15^N_Glx_ and δ^15^N_Phe_ (${\bar{\Delta }}^{15}N_{\text{Glx}–\mathrm{Phe}}$) for a set of *Cervus elaphus* individuals from the Angara, Little Sea and southern Upper Lena microregions (precise locations are indicated in Table S3), with an assumed TP_terr_ = 2. Based on the canonical model:

$$\delta^{15}N_{\mathrm{Glx}}=\delta^{15}N_{\mathrm{Phe}}+\beta +\mathrm{TDF}$$

Solving for *β* using the intercept from the fixed‐slope model (TDF = 7.6‰), we obtained:

$$\beta ={\bar{\Delta }}^{15}N_{\text{Glx}–\mathrm{Phe}}-7.6$$

This yielded a calibrated value of *β* = −7.28‰ ± 0.49‰. The resulting *β* is within the expected range for terrestrial producers (−6.6‰ ± 3.4‰ after Ramirez et al. 2021).

#### 2.3.6 Statistical Analyses

All statistical tests were performed using R 4.4.3 (R Core Team 2025). Normality was assessed using Shapiro–Wilk tests; due to non‐normal distributions and small sample sizes, non‐parametric tests were applied. Group differences were evaluated using Kruskal–Wallis tests (KW‐tests) with Dunn's post hoc tests comparisons. Linear regressions were used to explore relationships between isotopic variables, with model strength assessed using the Pearson correlation coefficient of determination (*R* ^2^) and associated *p*‐values derived from *t*‐tests.

#### 2.3.7 Dietary Clustering of Fish Species

Non‐metric multidimensional scaling (NMDS) was used to explore trophic structure among freshwater fish species based on a presence/absence matrix of 21 dietary categories compiled from published gut content data (www.fishbase.org; Froese and Pauly 2025, Table S4). Unsuitable for Euclidean distance‐based ordination methods, NMDS was therefore performed using the Jaccard dissimilarity index, appropriate for binary data. The analysis was implemented with the vegan package (Oksanen et al. 2025) in R 4.4.3 (R Core Team 2025).

To identify recurring dietary patterns, we applied k‐means clustering to the NMDS ordination scores. The optimal number of clusters was determined using both the Elbow and Silhouette criteria. Fish species were subsequently assigned to clusters, which were interpreted ecologically to facilitate comparison with isotopic data. Details of the successive analytical steps and associated figures are provided in the Supporting Information.

## 3 Results

All results are available in the Tables S3–S5. All discussed δ^66^Zn data are expressed as bone, either they are “bone‐equivalent” or “bone” values.

### 3.1 Data Quality

All analyzed samples exhibited well‐preserved collagen and all reference materials produced expected values for δ^66^Zn and ^87^Sr/^86^Sr values (SRM 1400, SRM 1486 and SRM 987; Table S5).

For CSIA‐AA, samples were run during 30 sessions. Analytical sessions were excluded when reference materials consistently yielded inaccurate δ^15^N values across multiple amino acids. When analytical issues were restricted to individual amino acids, only the corresponding data were excluded (e.g., Gly in two analytical sessions). Leucine values were discarded because the resolution of the peaks was insufficient (Figure S1). Peaks with amplitude lower than 100 mV were also excluded because the signal‐to‐background ratio becomes insufficient below this threshold, preventing reliable δ^15^N determination. The δ^15^N values for proline (Pro) and hydroxyproline (Hyp) were nearly identical (*R* ^2^ = 0.98, *y* = 0.967*x* + 0.169, *p* < 0.001; Figure S2B; O'Connell and Collins 2018).

### 3.2 Carbon, Nitrogen and Strontium Isotope Ratios of Human and Fauna Data

A total of 75 new measurements were generated for δ^13^C, δ^15^N, and ^87^Sr/^86^Sr. Among these, 20 carbon and nitrogen isotope values matched previously published data from the same individuals, enabling direct comparison (Table 2 and Figure S6). Additionally, 22 carbon and nitrogen isotope measurements were produced for animals that were not previously analyzed. For strontium isotopes, nine faunal specimens had newly obtained values (Table 2), which are consistent with the previously reported faunal ^87^Sr/^86^Sr ranges by Scharlotta and Weber (2012) for their respective microregions: 0.70811–0.71680 for Angara, 0.70694–0.74614 for the Little Sea, and 0.70849–0.71112 for the Upper Lena. Strontium isotope ratios were previously obtained on human M1, M2, M3, and bone samples for the three cemeteries. Thirteen human samples were reanalyzed for this study and gave matching values, except for the individual K14_1997.019 (Figure S6), for whom previously published measurements already showed high variability between the three teeth that were analyzed for this individual. This discrepancy between previous and new measurements may therefore reflect sampling of different areas of the dental crown.

**TABLE 2:** Average δ^66^Zn values for terrestrial herbivores and ^87^Sr/^86^Sr of fauna from various locations.

Microregion	Site	δ^66^Zn (‰)	SD (‰)	*n*	^87^Sr/^86^Sr	SD	*n*
Angara	Gorelyi Les	0.95	0.30	5			
	Kitoi				0.70868		1
	Lokomotiv	1.18	0.25	4			
	Ust’‐Ida I	1.26	0.05	1			
	Ust’‐Belaia	1.44	0.05	2			
	Ostrov Listvenichnyi	1.21	0.01	1			
	All sites	1.21	0.18	13			
SW Baikal	Shamanka II	1.12	0.21				
Little Sea	Berloga				0.71010	0.00080	2
	Sagan‐Zaba II	1.13	0.15	9	0.71427	0.00658	11
	Ityrkhei	1.09	0.04	2	0.71378	0.00239	2
	Mys Uiuga				0.71285	0.00017	2
	Ulan‐Khada				0.71330	0.00014	4
	All sites	1.12	0.14	11			
Upper Lena S	Makrushino	1.22	0.16	2			

*Note:* For sites represented by a single individual (*n* = 1), SD represents analytical variability among replicate measurements; for *n* > 1, SD represents inter‐individual variability.

Three canids from different sites (Mys Uiuga, Shamanka II, and Berloga; Figure 1) were analyzed. They may represent domestic dogs or commensal wolves. Their elevated δ^15^N values are consistent with a substantial contribution of aquatic‐derived protein to their diets, although bulk collagen δ^15^N values alone do not allow the relative contributions of fish, seal, and terrestrial resources to be quantified. Similar isotopic patterns have been reported for prehistoric dogs from the Cis‐Baikal region, where dogs often occupied dietary niches closely associated with local human populations and consumed aquatic resources (Losey et al. 2013, 2011). This pattern is consistent with the commensal relationship between humans and domestic dogs that developed through domestication (Serpell 2021). As expected for the region, herbivore values indicate a diet relying on C_3_ plants.

### 3.3 Calculated and Theoretical Trophic Positions of Lake Baikal Fauna

Terrestrial herbivores were used to calibrate the *β* value applied in TP_AA_ estimation (*β* = −7.28‰ ± 0.49‰, mean ± SD, *n* = 10), which is consistent with published values for terrestrial food webs (Ramirez et al. 2021). Consequently, terrestrial herbivores show, by definition, trophic levels (TP_AA_) centered around TP_terr_ = 2 (mean ± SD: 2 ± 0.2, mean ± SD, *n* = 10, Figure 2). In fish, TP_bulk_ (calculated from δ^15^N_bulk_), and TP_AA_ (TP_aq_, calculated from Δ^15^N_Glx–Phe_) generally align with TP_theo_, although some taxa show underestimation relative to TP_theo_ (Table 3). Among seals, TP_AA_ values averaged 3.17 (± 0.21, *n* = 14) and TP_bulk_ = 4.24 (±0.1, *n* = 3). TP_theo_ is estimated at four for Baikal seals (Yoshii et al. 1999).

**FIGURE 2:**
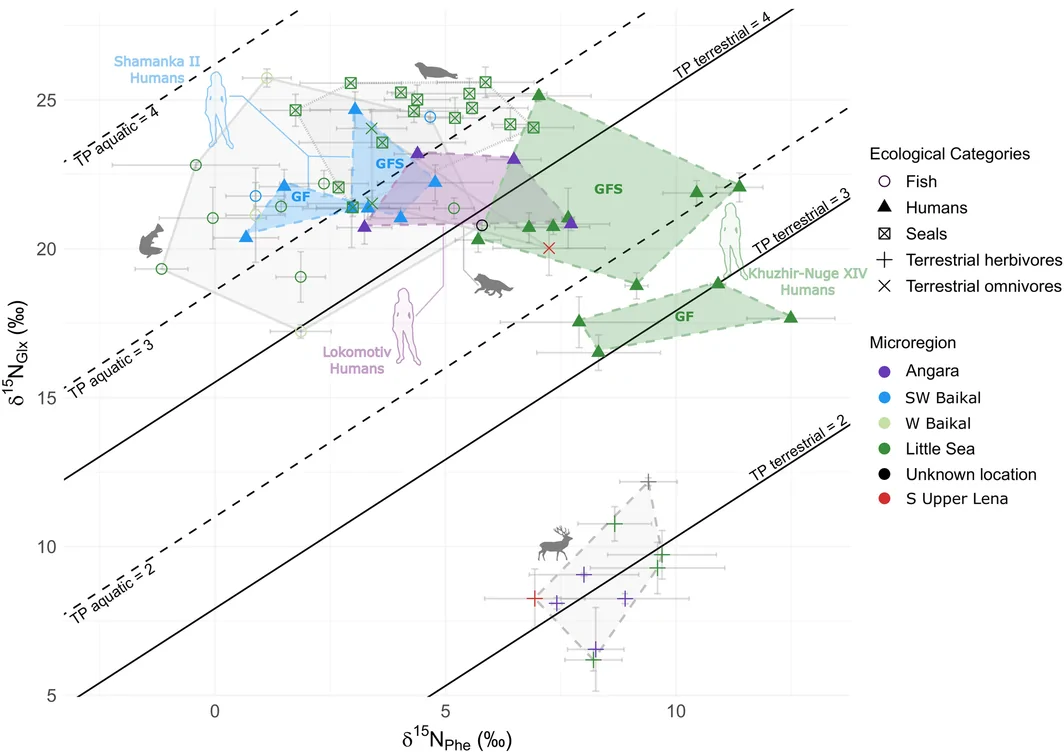
Relationship between δ^15^N for Glutamic acid (Glx) and Phenylalanine (Phe) values and Trophic Position (TP) lines in Cis‐Baikal. The plot shows the relationship between δ^15^N_Glx_ and δ^15^N_Phe_ values for ecological categories (fish, humans, seals, terrestrial herbivores, and omnivores). Each point represents an individual, colored by microregion and shaped according to the ecological category, as detailed in the legend. The dashed and solid lines represent trophic positions (TP) for aquatic and terrestrial food webs respectively (TP = 2, 3, and 4 from bottom to top). The SWB‐GF and SWB‐GFS groups correspond to the dietary classification proposed in this study based on the δ^66^Zn distribution of Shamanka II individuals and comparison with the previously established GF and GFS groups from Khuzhir‐Nuge XIV (Sections 3.4.2 and 4.2). The “Unknown location” category refers to a fish specimen documented only as originating from Lake Baikal, for which no more precise assignment was possible.

**TABLE 3:** Trophic position (TP) estimates for fish species.

Species	TP_theo_ mean (±SD)	TP_bulk_ mean (±SD)	TP_AA_ mean (± SD)	Dietary cluster
*Abramis brama*	3.1 (±0.1)	—	—	(1) Opportunistic Benthic Omnivores
*Acipenseridae*	3.3 (±0.2)	3.6 (*n* = 1)	3.2 (*n* = 1)	(1) Opportunistic Benthic Omnivores
*Brachymystax lenok*	3.8 (±0.3)	—	—	(2) Opportunistic Benthic Predators
*Comephorus dybowskii*	3.4 (±0.5)	—	—	(5) Low‐TP selective feeders
*Coregonus autumnalis*	3.7 (±0.2)	—	—	(1) Opportunistic Benthic Omnivores
*Coregonus baicalensis*	3.4 (±0.3)	3.6 (±0.2)	—	(5) Low‐TP selective feeders
*Coregonus lavaretus*	3.1 (±0.0)	—	—	(4) Opportunistic Zoo‐Benthivores
*Coregonus migratorius*	3.4 (±0.3)	2.7 (*n* = 1)	—	(5) Low‐TP selective feeders
*Cottocomephorus grewingkii*	3.1 (±0.0)	—	—	(5) Low‐TP selective feeders
*Esox lucius*	4.1 (±0.4)	3.4 (±0.09)	2.9 (±0.3)	(3) Apex Benthic Piscivores
*Gobio gobio*	3.1 (±0.4)	—	—	(1) Opportunistic Benthic Omnivores
*Hucho taimen*	4.5 (±0.3)	—	—	(3) Apex Benthic Piscivores
*Leocottus kesslerii*	3.2 (±0.3)	—	—	(5) Low‐TP selective feeders
*Leuciscus idus*	3.8 (±0.6)	3.1 (±0.57)	2.6 (±0.1)	(2) Opportunistic Benthic Predators
*Leuciscus leuciscus*	2.9 (±0.1)	—	—	(1) Opportunistic Benthic Omnivores
*Lota lota*	3.8 (±0.2)	3.6 (*n* = 1)	3.6 (*n* = 1)	(2) Opportunistic Benthic Predators
*Perca fluviatilis*	4.4 (±0.0)	3.1 (±0.2)	3.3 (*n* = 1)	(4) Opportunistic Zoo‐Benthivores
*Rutilus rutilus*	3.0 (±0.0)	3.0 (±0.2)	3.0 (±0.3)	(1) Opportunistic Benthic Omnivores
*Thymallus arcticus*	3.3 (±0.2)	—	—	(4) Opportunistic Zoo‐Benthivores
*Thymallus baicalensis*	3.5 (±0.5)	—	—	(5) Low‐TP selective feeders
*Thymallus baicalensis brevipinnis*	3.2 (±0.4)	3.1 (±0.4)	3.5 (±0.3)	(5) Low‐TP selective feeders
*Thymallus baicalensis ssp*.	3.4 (±0.4)	—	—	(5) Low‐TP selective feeders

*Note:* TP_theo_ corresponds to theoretical values from the literature; TP_bulk_ refers to TP calculated from bulk collagen δ^15^N using the Lake Baikal pelagic food‐web equation of Yoshii et al. (1999), see Section 2.3.5; TP_AA_ indicates trophic position calculated from amino acid–specific nitrogen isotopes (CSIA‐AA). Dietary clusters identified using NMDS and k‐means clustering applied to a binary matrix of fish dietary traits (see Sections 2.3.7 and 3.4.1). Data are expressed as mean ± SD. Missing values (—) indicate unavailable data.

Looking at the distribution of δ^15^N_Phe_ values of fauna and human tissues (Figure 2), a clear lacustrine baseline signal is formed by fish (1.88‰ ± 2.15‰, *n* = 13) and seals (4.44‰ ± 1.53‰, *n* = 14), and is also expressed in Shamanka II humans, with low δ^15^N_Phe_ values (2.9‰ ± 1.41‰, *n* = 7). In contrast, terrestrial herbivores (7.63‰ ± 2.07‰, *n* = 13) cluster around higher δ^15^N_Phe_ values, forming a terrestrial baseline pole. Within Khuzhir‐Nuge XIV, GF individuals display the highest mean δ^15^N_Phe_ values (9.9‰ ± 2.18‰, *n* = 4), whereas GFS individuals show slightly lower values (8.19‰ ± 1.95‰, *n* = 8), consistent with a greater influence of aquatic resources on their dietary baseline. Humans from Lokomotiv (Angara) are positioned between these two opposites (5.46‰ ± 2.02‰, *n* = 4), though the lack of δ^15^N_Phe_ data for local freshwater fish prevents a definitive baseline assignment; their values remain intermediate between the lacustrine and terrestrial poles, potentially reflecting a mixed resource base.

CSIA‐AA‐derived trophic position estimates (TP_AA_) further contextualize these distributions (Figure 2, Table 3). Hereafter, TP_aq_ and TP_terr_ refer to TP_AA_ estimates calculated using the aquatic and terrestrial *β* values, respectively. Fishes and seals align predominantly above the TP_aq_ = 3 line. The trophic position of humans was not calculated because of the mixing of terrestrial and aquatic resources. However, Shamanka II humans are distributed around the TP_aq_ = 3 line (Figure 2), with two GF and one GFS individuals plotting above the line, while three GFS and one GF individuals fall below it. GFS individuals from Khuzhir‐Nuge XIV display more scattered signatures between the TP_aq_ = 3 and TP_terr_ = 3 lines, as expected for diets combining resources from food webs with distinct isotopic baselines. In contrast, GF individuals from Khuzhir‐Nuge XIV group around the TP_terr_ = 3 line or slightly below and exhibit high δ^15^N_Phe_ values, consistent with diets dominated by terrestrial animal protein. One young GF individual from Khuzhir‐Nuge XIV (K14_1998.037.02) falls slightly below the TP_terr_ = 3 line, suggesting a greater contribution of plant‐derived proteins than in the rest of the group, although terrestrial animal resources likely remained the main source of dietary protein.

### 3.4 Zinc Isotope Ratios Variation and Trophic Structure in Lake Baikal

#### 3.4.1 Ecological Clustering and Zn Isotope Variability Among Fish

Zinc isotope ratios in Lake Baikal fish span a wide range of values (0.20‰ to 1.68‰; mean 0.80‰ ± 0.29‰, *n* = 76). Differences among locations show low statistical significance (*χ* ^2^ = 10.5, df = 7, *p* = 0.16, KW‐test). Despite expectations of a decreasing trend in δ^66^Zn values with increasing trophic level, no correlation is observed with TP_theo_ (Table S1) or with TP_bulk_. However, fish displayed significantly higher δ^66^Zn values with lower TP_AA_, although this relationship is based on a smaller number of species (*n* = 7), compared with TP_bulk_ (*n* = 9) and TP_theo_ (*n* = 22).

Using NMDS and k‐means clustering on a presence/absence dietary matrix (Table S4), five distinct trophic clusters were identified among the 76 fish specimens. These clusters, defined independently of isotopic data, were labeled based on feeding strategies: (1) Opportunistic Benthic Omnivores, (2) Opportunistic Benthic Predators, (3) Apex Benthic Piscivores, (4) Opportunistic Zoo‐Benthivores, and (5) Low‐TP Selective Feeders (Table 3 and Table S4). Significant differences in δ^66^Zn values across these clusters are recorded (KW‐test, *χ* ^2^ = 23, df = 4, *p* = 1.3 × 10^−4^), indicating that the nature of the food consumed is associated to specific Zn isotope values. Apex Benthic Piscivores consist of two species *Hucho taimen* and *Esox Lucius* . Notably, the two clusters with the highest dietary richness (number of prey types consumed)—opportunistic zoo‐benthivores (mean dietary richness = 13.7) and benthic omnivores (13.2)—also display high δ^66^Zn variability (SD = 0.30‰ and 0.27‰, respectively). In contrast, low‐TP selective feeders, which exhibit the narrowest dietary niche (mean = 6.7 prey types), show markedly the lowest δ^66^Zn variability (SD = 0.17‰). A positive linear trend was identified between dietary richness and δ^66^Zn standard deviation across clusters (*R* ^2^ = 0.65, *p* = 0.04), supporting the hypothesis that broader dietary spectra are associated with increased isotopic variability. Taxa with broader trophic spectra (mean dietary richness ≥ 14) exhibit moderate intra‐specific δ^66^Zn standard deviations (ranging from 0.09‰ to 0.32‰), while more specialized taxa (e.g., richness = 9) show reduced isotopic variability (SD = 0.05‰ to 0.15‰). The extreme specialist *Comephorus dybowskii* (dietary richness = 1) displays the lowest δ^66^Zn variability (SD = 0.03‰). Interestingly, Δ^15^N_Glx–Phe_ values do not differ significantly across dietary clusters (KW‐test, *χ* ^2^ = 5.71, df = 4, *p* = 0.22), a pattern that may reflect the smaller number of fish specimens analyzed by CSIA‐AA compared to δ^66^Zn.

#### 3.4.2 Zinc Isotope Variability Among Mammals

Terrestrial herbivorous taxa (e.g., bovids, cervids, equids, and marmots) exhibit higher δ^66^Zn values (1.18‰ ± 0.22‰) than other terrestrial animals, which is expected and was previously observed in modern and prehistoric food webs (Bourgon 2022; Guiserix et al. 2024; Jaouen, Beasley, et al. 2016; Jaouen et al. 2022; Martin and Jaouen 2024; Pederzani et al. 2024). As has often been observed in open environments (Guiserix et al. 2024; Jaouen, Beasley, et al. 2016; Jaouen et al. 2022; Pederzani et al. 2024), grazing species (*Bos* sp., *Equus* sp.) tend to exhibit higher values than mixed feeders or browsers ( *Cervus elaphus* , *Capreolus* sp.). *Sus scrofa* , conventionally classified here as an omnivore, displays similarly high and more variable δ^66^Zn values (1.25‰ ± 0.37‰, *n* = 3). *Carnivora* taxa, both terrestrial (canids and *Ursus arctos* ; 0.69‰ ± 0.19‰) and lacustrine (Baikal seals; 0.60‰ ± 0.11‰), show lower δ^66^Zn values that overlap with the human range (0.63‰ ± 0.16‰, Figure 3).

**FIGURE 3:**
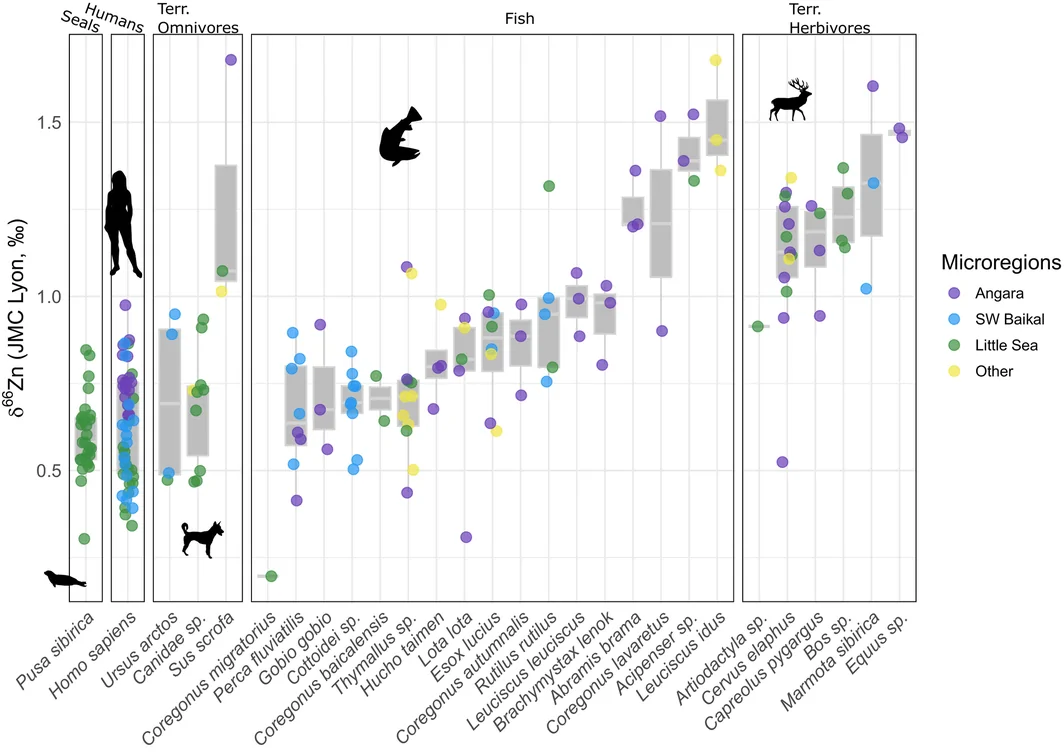
General box plot of δ^66^Zn in Cis‐Baikal. Distribution of δ^66^Zn values within ecological groups. Values from the same taxon (species to suborder) are plotted within the same box plot. For visual clarity, closely related taxa were grouped in this overview figure: The three *Cottoidei* species ( *Comephorus dybowskii* , *Cottocomephorus grewingkii* , and *Leocottus kesslerii* ) were represented as *Cottoidei* sp., *Thymallus* taxa as *Thymallus* sp., and canids under a common *Canidae* category. Precise taxonomic level identifications and associated isotopic data are provided in Table S3.

Human dietary groups show marked differences in δ^66^Zn values. At Khuzhir‐Nuge XIV, Little Sea (LS), GFS humans exhibit lower values (LS‐GFS, δ^66^Zn = 0.48‰ ± 0.08‰, *n* = 15) than both LS‐GF individuals (δ^66^Zn = 0.78‰ ± 0.07‰, *n* = 4) and individuals from Lokomotiv (δ^66^Zn = 0.77‰ ± 0.08‰, *n* = 16, Figure 4). Shamanka II individuals in SW Baikal (SWB) span the full δ^66^Zn range observed between LS‐GF and LS‐GFS individuals. As discussed in detail in Section 4.2, SWB individuals were therefore subdivided, by analogy with the previously established Little Sea dietary groups, into Game + Fish consumers (SWB‐GF; δ^66^Z*n* = 0.68‰ ± 0.11‰, *n* = 8) and Game + Fish + Seal consumers (SWB‐GFS; δ^66^Z*n* = 0.46‰ ± 0.05‰, *n* = 7), using a threshold of 0.56‰, corresponding to the mean + 1 SD of the LS‐GFS δ^66^Zn distribution (0.48‰ ± 0.08‰; Figure 4 and Table 4). Individuals sampled on first molars or canine tend to exhibit higher δ^66^Zn values than other permanent teeth (Figure 4). This pattern is consistent with previous observations (Jaouen et al. 2020), and has been interpreted as the signature of breastmilk consumption.

**FIGURE 4:**
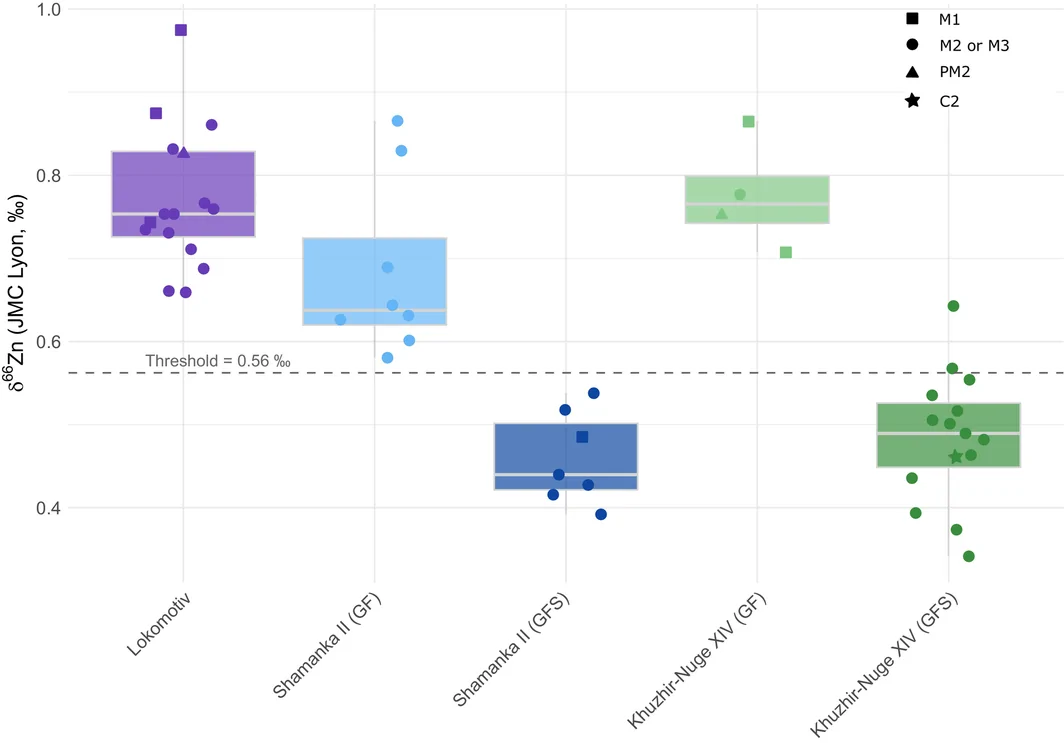
Threshold‐based differentiation of δ^66^Zn values among human individuals. The threshold (mean + 1 SD) was defined from Khuzhir‐Nuge XIV GFS individuals and is applied to distinguish Shamanka II individuals consuming Game + Fish + Seals (SWB‐GFS) versus primarily Game + Fish (SWB‐GF).

**TABLE 4:** Zinc, nitrogen, strontium and carbon isotope values for each dietary group of the three sites explored in this study.

Microregion (cemetery; abbreviation)	Dietary group	${\bar{\delta }}^{66}\mathrm{Zn}$ (‰, mean ± SD)	${\bar{\Delta }}^{15}N_{\text{Glx}–\mathrm{Phe}}$ (‰, mean ± SD)	^87^Sr/^86^Sr (mean ± SD)	${\bar{\delta }}^{15}N_{\text{dentin}}$ (‰, mean ± SD)	${\bar{\delta }}^{13}C_{\text{dentin}}$ (‰, mean ± SD)	References
Little Sea (Khuzhir‐Nuge XIV; LS)	LS‐GFS (Game‐Fish‐Seal consumers)	0.48 ± 0.08 (*n* = 15)	13.14 ± 2.64 (*n* = 8)	0.7123 ± 1.00 × 10^−3^ (*n* = 15)	15.12 ± 0.90 (*n* = 15)	−17.70 ± 0.61 (*n* = 15)	Haverkort et al. (2008); Weber et al. (2003), Weber et al. (2021); Weber et al. (2016b)
Little Sea (Khuzhir‐Nuge XIV; LS)	LS‐GF (Game‐Fish consumers)	0.78 ± 0.07 (*n* = 4)	7.73 ± 1.87 (*n* = 4)	0.7108 ± 8.60 × 10^−4^ (*n* = 4)	11.55 ± 0.79 (*n* = 4)	−19.02 ± 0.28 (*n* = 4)	
SW Baikal (Shamanka II; SWB)	SWB‐GFS (Game‐Fish‐Seal consumers)	0.46 ± 0.05 (*n* = 7)	18.62 ± 2.09 (*n* = 4)	0.7130 ± 1.68 × 10^−3^ (*n* = 7)	14.76 ± 0.48 (*n* = 7)	−15.99 ± 0.48 (*n* = 7)	Weber et al. (2016a); Weber et al. (2016b)
SW Baikal (Shamanka II; SWB)	SWB‐GF (Game‐Fish consumers)	0.68 ± 0.11 (*n* = 8)	19.44 ± 1.29 (*n* = 3)	0.7120 ± 8.90 × 10^−4^ (*n* = 8)	14.00 ± 0.80 (*n* = 8)	−16.15 ± 0.70 (*n* = 8)	
Angara (Lokomotiv)	—	0.77 ± 0.08 (*n* = 16)	16.47 ± 2.43 (*n* = 4)	0.7105 ± 6.30 × 10^−4^ (*n* = 16)	14.78 ± 0.67 (*n* = 16)	−15.29 ± 0.55 (*n* = 16)	Weber et al. (2016a)

*Note:* GF and GFS differentiation for Shamanka II cemetery is detailed in Section 3.4.2.

Prehistoric human ^87^Sr/^86^Sr and δ^66^Zn values from the Cis‐Baikal dataset negatively correlate (Figure S3), consistent with differences among microregions characterized by distinct geological substrates and dietary practices. This correlation is not visible for terrestrial herbivores whose δ^66^Zn values are comparable across locations (Table 2 and Figure S3).

### 3.5 Comparative Analysis of Trophic Level Indicators

Linear regressions confirmed a negative relationship between δ^66^Zn and TP_AA_ across fishes, in Little Sea humans + terrestrial fauna, and in SWB‐GFS + seals + fish (Table S1).

Principal Component Analysis (PCA) incorporating δ^66^Zn and other δ^15^N_AA_ separates samples along PC1 (59.37% of the total variance), primarily associated with trophic variation and aquatic vs. terrestrial gradients, and PC2 (19.96%), primarily associated with differences in ecological baselines (Figure 5). PC1 is mainly driven by trophic amino acids (δ^15^N_Ala_, 22.2%, δ^15^N_Val_, 21.1%; δ^15^N_Glx_, 20.3%; and δ^15^N_Asx_, 19.1%), whereas PC2 is dominated by δ^15^N_Phe_ (55.7%) and δ^15^N_Gly_ (36.8%). δ^66^Zn contributes modestly to both components (9.89 and 2.10%, respectively). Importantly, the only strictly source amino acid measured, δ^15^N_Phe_, together with δ^15^N_Gly_—a non‐essential amino acid known to behave similarly to source amino acids—aligns primarily along PC2, highlighting the different ecological baselines. SWB‐GF and SWB‐GFS individuals separate primarily along PC1 (trophic dimension), while LS‐GF and LS‐GFS groups are differentiated along both PC1 and PC2 (combined trophic and baseline effect, Figure 5).

**FIGURE 5:**
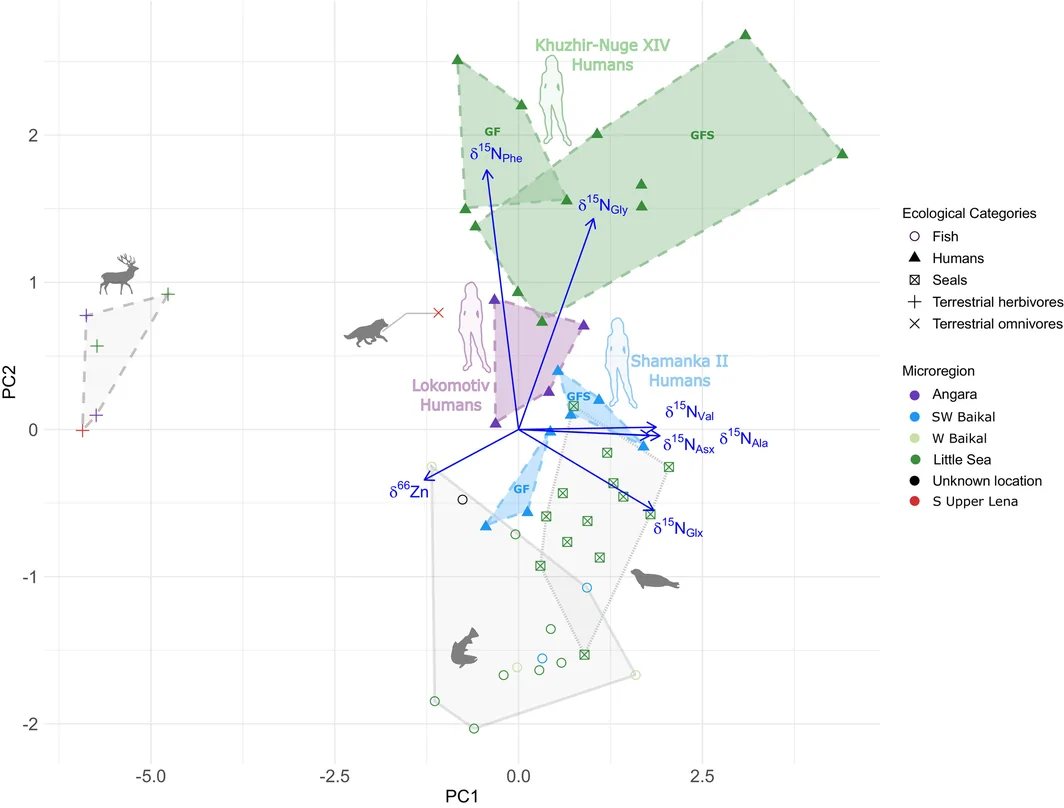
Individual distribution and variable contribution. The plot presents the results of a Principal Component Analysis (PCA) showing the distribution of individuals (single points) and the contribution of variables (blue arrows) to the first two principal dimensions (PC1 and PC2). Arrows indicate the direction and relative contribution of each variable (e.g., δ^15^N_Phe_, δ^15^N_Glx_, δ^66^Zn) to the PCA dimensions.

With respect to the Δ^15^N_Glx–Phe_ trophic proxy, SWB‐GF and SWB‐GFS individuals display similar values (19.44‰ ± 1.29‰, *n* = 3 and 18.62‰ ± 2.09‰, *n* = 4, respectively), whereas the two Little Sea groups are clearly differentiated (LS‐GFS: 13.14‰ ± 2.64‰, *n* = 8; LS‐GF: 7.73‰ ± 1.87‰, *n* = 4; Figure 2, Table 4).

Bulk collagen δ^15^N–δ^13^C values distinguish LS‐GF and LS‐GFS groups, whereas SWB‐GF and SWB‐GFS individuals show greater overlap. Nevertheless, when classified on the basis of δ^66^Zn, SWB individuals display a distribution comparable in bulk δ^15^N–δ^13^C values to that observed in LS, with GF individuals generally plotting toward lower δ^15^N and δ^13^C values than GFS individuals (Figure S5).

## 4 Discussion

### 4.1 Zinc Isotope Ratios and Trophic Structure of Lake Baikal

Unlike terrestrial mammals, fishes do not show a clear relationship between their δ^66^Zn values and their theoretical trophic position. Nor do they show a clear relationship with trophic positions estimated from bulk collagen δ^15^N values (TP_bulk_). This indicates that, within the Lake Baikal fish dataset, δ^66^Zn does not behave as a simple linear proxy for trophic position. Still, it is possible that these fish actually have different trophic positions in Lake Baikal compared to other places, since their calculated TP_AA_ differ from the theoretical ones (Table 3). TP_theo_ values are derived from published ecological information and may not fully represent the trophic positions of the sampled populations within the Lake Baikal food web. In addition, TP_bulk_ is sensitive to variation in δ^15^N baselines among habitats and food webs. By contrast, trophic positions estimated from δ^15^N_AA_ data (TP_AA_) better separate baseline variation from trophic enrichment. This hypothesis is supported by the fact that δ^66^Zn values and TP_AA_ correlate (Table S1). Although this correlation is based on a limited number of fish specimens, it suggests that δ^66^Zn retains a trophic component in fish. However, the trophic clustering reveals consistent ecological patterns across fish taxa suggesting that δ^66^Zn values reflect both trophic position and the δ^66^Zn composition, Zn content, and tissue composition of consumed prey, rather than the trophic level alone (Bourgon et al. 2024). The interpretation of such dietary effects nevertheless requires independent ecological, archeological, or isotopic information to characterize the Zn isotope composition and Zn concentration of potential prey. In the absence of distinguishable prey signatures or complementary proxies, δ^66^Zn values alone may not resolve precise dietary differences among fish taxa. Due to zinc isotope fractionation in vertebrate bodies, their muscles tend to have much lower Zn isotope ratios than bones. In other words, the documented link between Zn isotope ratios and trophic positions appears to be more clearly expressed in cases of incomplete consumption of vertebrate prey, with preferential intake of soft tissues rather than skeletal elements. This could explain why the trophic structure of freshwater food webs is not clearly resolved here, given that many fish species consume invertebrates or whole vertebrate prey. Among fish, higher δ^66^Zn values are observed in opportunistic benthic omnivores and predators, while lower values are characteristic of low‐trophic, selective feeders (Figure 6). The smaller‐than‐expected δ^66^Zn offset between seals and fishes is consistent with the feeding ecology of Baikal seals, whose diet includes small pelagic fishes (*Cottoidei*), as well as the endemic pelagic amphipods *Macrohectopus branickii*. Stable isotope mixing models have estimated contributions of approximately 54% pelagic sculpins and 46% *M. branickii* to the diet of Baikal seals (Yoshii et al. 1999), while direct observations have subsequently confirmed intensive predation on M. branickii (Watanabe et al. 2020). These prey are ingested whole, which reduces isotopic offsets arising from selective tissue intake by incorporating skeletal components enriched in heavy Zn isotopes (McCormack et al. 2024) and controlled feeding experiments indicate that consumer tissue δ^66^Zn is primarily determined by the isotopic composition of the dietary Zn pool (Bourgon et al. 2024). However, the magnitude of this effect cannot currently be quantified for Baikal seals because neither δ^66^Zn nor Zn concentrations are available for *M. branickii*. Consequently, the relative contributions of fish and amphipods to the dietary Zn pool may differ from their estimated contributions to dietary biomass and cannot be reconstructed from the available δ^66^Zn data alone. It should also be noted that most fish analyzed are modern and therefore may not fully reflect prehistoric Lake Baikal ecosystems. The number of prehistoric specimens available for each fish taxon is too limited to allow a reliable statistical comparison between modern and prehistoric samples. This limitation is particularly relevant in the Angara microregion where hydroelectric dam construction substantially altered river connectivity and fish communities during the 20th century (Weber et al. 2011). This limitation should be considered when interpreting modern fish as proxies for prehistoric populations.

**FIGURE 6:**
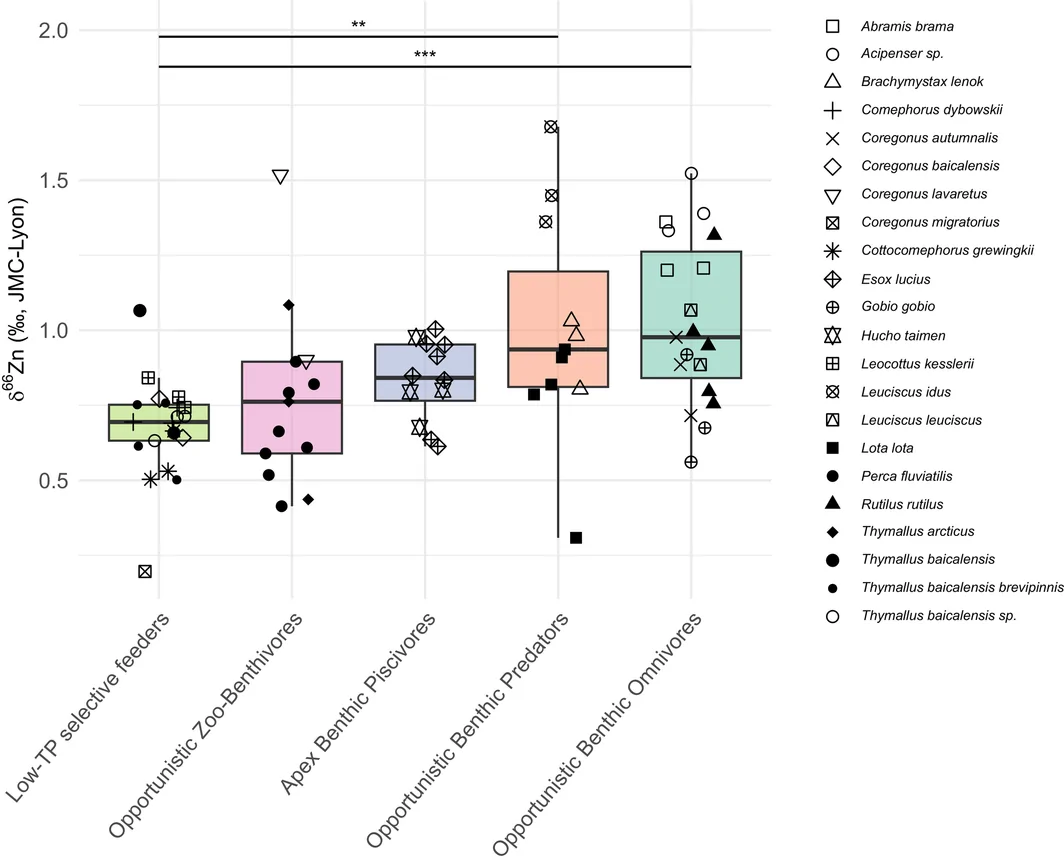
Distribution of δ^66^Zn values across fish dietary clusters. Box plots represent the median and interquartile range for each cluster. Each point corresponds to an individual fish, with species identified by distinct symbols (see legend below). Significant pairwise differences from a Dunn post hoc test are indicated above the plot: ***p ≤ 0.001, **p ≤ 0.01.

The lower TP_AA_ values observed for Baikal seals relative to their expected TP_theo_ likely reflect the substantial contribution of pelagic low‐ to mid‐trophic prey to their diet, particularly the endemic amphipod *Macrohectopus branickii*, which was not analyzed in the present study. This species constitutes a non‐negligeable food resource for Baikal seals, which exhibit high foraging rates on small pelagic crustaceans (Watanabe et al. 2020). Such reliance on pelagic amphipods may reduce trophic estimates based on Δ^15^N_Glx–Phe_, resulting in TP_AA_ values lower than those inferred from bulk isotopic data or theoretical expectations (Karnaukhov et al. 2021; Yoshii et al. 1999). In addition, the discrepancy between CSIA‐AA‐ and bulk‐based trophic estimates for seals could also reflect uncertainty in the Glx–Phe nitrogen TDF applicable to carnivores, as recent evidence suggests that lower values than the conventional 7.6‰ may be appropriate in some cases (Krajcarz et al. 2025).

Ursids and canids have zinc isotopic values similar to those of humans, although on average slightly higher. This is consistent with diets including animal resources, but CSIA‐AA data are available for only two canids and no ursids in the present study which limits more precise interpretation of their trophic position. Clusters are noted in the distribution of Zn isotopic data for canids, which could indicate the consumption of fish or seals by some and therefore potentially the presence of domestic dogs among the sampled canids, as previously interpreted for Cis‐Baikal canids (Losey et al. 2011, 2013).

*Sus scrofa* , although conventionally classified as an omnivore, exhibits high δ^66^Zn values comparable to or exceeding those of terrestrial herbivores. This pattern is consistent with a predominantly plant‐based diet, as commonly documented for wild boar despite their opportunistic feeding behavior (Ballari and Barrios‐García 2014; Wilman et al. 2014). Bourgon et al. (2025) proposed that the relatively high δ^66^Zn values observed in *Sus* sp. may partly reflect the consumption of underground plant organs through rooting behavior. This hypothesis is consistent with the synthesis of plant Zn isotope data by Méjean et al. (2026), which shows that roots and tubers display among the highest δ^66^Zn values of plant tissues. The variability observed among the three *Sus scrofa* individuals may reflect their dietary opportunism, with the two lower δ^66^Zn values potentially indicating a greater contribution of animal resources, although the limited sample size precludes further interpretation. Similarly, the apparent grouping of *Ursus arctos* δ^66^Zn values should be interpreted cautiously given the small number of individuals. Their broad isotopic range may nevertheless be consistent with the dietary plasticity expected for an omnivorous taxon.

### 4.2 New Insights Into Kitoi and Glazkovo Subsistence Strategies

The combination of δ^15^N of amino acids and δ^66^Zn allows a refined assessment of the subsistence strategies of the three hunter‐gatherer groups studied. Notably, the introduction of Zn isotopes offers new evidence that both corroborates and refines these earlier interpretations. Individuals previously identified as GFS at Khuzhir‐Nuge XIV exhibit systematically lower δ^66^Zn values than GF individuals, which is consistent with their seal consumption. Importantly, this new isotopic pattern provides a reference framework that can be extended to other microregions. At Shamanka II, human δ^66^Zn values span a similarly wide range, with the lower part of the distribution strongly overlapping LS‐GF values and the higher values extending into the range observed for LS‐GFS individuals. This similarity with the Little Sea dataset may suggest the existence of two dietary groups within the SWB population. By analogy with the Little Sea framework, individuals with higher δ^66^Zn values are labeled as SWB‐GF, whereas those exhibiting markedly lower δ^66^Zn values are classified as SWB‐GFS (Section 3.4.2, Table 4 and Figure 4). The markedly lower δ^66^Zn values of GFS individuals are therefore interpreted as indicative of seal consumption. This discrepancy can be explained by differences in Zn bioavailability among food types. Baikal seals' muscles contain 39–45 mg Zn·kg^−1^ wet weight (Watanabe et al. 2020), terrestrial herbivores 19–64 mg Zn·kg^−1^ (Falandysz et al. 2005), and Baikal fish only ~2.4 mg Zn·kg^−1^ (Kuznetsova et al. 2002). Given that seal muscle contains 15 to 20 times more Zn than fish muscle, even modest contributions of seal meat to the diet can disproportionately influence the consumer's Zn isotope composition. In other words, Zn isotopes are more sensitive to seal consumption than nitrogen or carbon isotopes. This pattern of seal consumption based on bulk collagen δ^15^N has been recently discussed by van der Haas (2023, 167), who pointed out that elevated bulk collagen δ^15^N values in some LS‐GFS individuals could be produced without any seal consumption, since high‐trophic fish such as perch and pike can yield similarly high δ^15^N_bulk_ signatures. In contrast, δ^66^Zn provides additional discriminatory information. Although perch can exhibit low δ^66^Zn values overlapping those of seals, pike shows substantially higher values. Moreover, fish muscle contains much less Zn than mammal muscle, implying that a fish‐based diet would be required for low‐δ^66^Zn fish to exert a comparable influence on consumer δ^66^Zn. Such a scenario is difficult to reconcile with the terrestrial δ^15^N_Phe_ baseline observed in LS individuals and with the broader archeological evidence for subsistence system including terrestrial game. The intake of seals—given their extremely low δ^66^Zn values and much higher Zn concentrations—provides the most parsimonious explanation for the markedly low δ^66^Zn values observed in both GFS groups (Weber et al. 2025, 2016a).

The similarity of Δ^15^N_Glx–Phe_ mean values between SWB‐GF and SWB‐GFS individuals indicates that this proxy reflects the consumption of high‐trophic‐level resources at Shamanka II, rather than the specific contribution of seals. Several fishes consumed in SW Baikal, such as pike, occupy trophic positions comparable to those of Baikal seals, limiting the discriminative power of Δ^15^N_Glx–Phe_ in this context. In contrast, the clear separation observed between LS‐GF and LS‐GFS reflects a dietary system in which aquatic resource consumption by some individuals produces marked increases in Δ^15^N_Glx–Phe_ within populations that otherwise rely primarily on terrestrial protein sources, as indicated by their δ^15^N_Phe_ baseline. At Shamanka II, the overall contribution of aquatic resources is higher, resulting in uniformly elevated δ^15^N_bulk_ values and reduced isotopic contrast in bulk δ^15^N–δ^13^C space. The emergence of a GF vs. GFS differentiation at Shamanka II only after classification based on δ^66^Zn values highlights the added value of zinc isotopes in this context. By capturing differences in resource type linked to zinc bioavailability rather than trophic level alone, δ^66^Zn enables the identification of seal consumption where nitrogen‐based proxies reach their interpretive limits. Taken together, the combined isotopic proxies indicate that fish played a more important role in the diet of the SW Baikal individuals analyzed here than in those from the Little Sea.

The other Kitoi individuals considered in this study, from Lokomotiv, exhibit high δ^66^Zn and intermediate δ^15^N_Phe_ values. Although high Zn isotope ratios could potentially reflect local geological influences associated with limestone substrates (Figure 1, Table 1), no consistent regional pattern is observed among Angara fauna which exhibit similar Zn isotope ratios to those from Little Sea or SW Baikal (Table 2). The convergence of Lokomotiv human values toward midpoints between terrestrial and lacustrine poles could indicate mixed dietary inputs, but without robust riverine baselines for comparison, this remains inconclusive. Their high δ^66^Zn values and lower Δ^15^N_Glx–Phe_ (Table 4) argue against regular seal consumption in this group, reinforcing previous interpretations of an increased use of fish from the nearby river (Weber et al. 2021). The three fish species on which this population relied more have intermediate δ^66^Zn values compared to the rest of the fauna (Figure 3), which could suggest a trophic level spacing of +0.045‰ ± 0.058‰ between them and the humans. This positive offset is higher than expected. Theoretically, consumers should exhibit lower δ^66^Zn values than their diet, due to preferential retention of light isotopes in soft tissues (Jaouen, Beasley, et al. 2016; Martin and Jaouen 2024). The absence of this anticipated depletion suggests that other dietary sources with higher δ^66^Zn values may have contributed to the observed signal. The intermediate δ^15^N_Phe_ values of Lokomotiv individuals are consistent with mixed terrestrial and aquatic protein inputs, rather than exclusive reliance on riverine fish. Archeological evidence indicates that the hunting of large cervids formed a consistent component of subsistence during the Kitoi period, alongside the exploitation of aquatic resources (Weber 2020; Weber et al. 2021). In particular, even limited consumption of terrestrial herbivores such as cervids—whose δ^66^Zn values are markedly high and whose muscle tissue contains approximately 10 times more zinc than fish (Falandysz et al. 2005; Kuznetsova et al. 2002)—could have significantly influenced the consumer's overall δ^66^Zn signature. Thus, terrestrial game consumption provides a plausible explanation for the unexpectedly high δ^66^Zn values observed in Lokomotiv individuals alongside their intermediate δ^15^N_Phe_ values.

Moving on to the Glazkovo hunter‐gatherers from Khuzhir‐Nuge XIV, compound‐specific nitrogen isotope analysis enables the separation of baseline origin and trophic level, using δ^15^N_Phe_ as a proxy for the isotopic composition of the primary protein source, and Δ^15^N_Glx–Phe_ as a trophic position estimator. Within a single food chain, δ^15^N_Phe_ values are similar from one trophic step to the next (Chikaraishi et al. 2009; Ohkouchi et al. 2017; Ramirez et al. 2021). In the present dataset this is the case for fish, seals, and Shamanka II humans, suggesting a shared lacustrine baseline. Consistent with previous literature, this records the primary source of consumed proteins. In contrast, terrestrial herbivores and several Khuzhir‐Nuge XIV individuals of the GF dietary group exhibit markedly higher δ^15^N_Phe_ values, consistent with a terrestrial baseline. Among the Khuzhir‐Nuge XIV humans who present high δ^15^N_Phe_ values typical of terrestrial resources, one young GF individual (aged 14–17 years; K14_1998.037.02) displays relatively low δ^15^N_Glx_ and consequently the lowest Δ^15^N_Glx–Phe_ value of all individuals studied here, placing this individual below the expected TP_terr_ = 3 line for terrestrial game consumers. Given that collagen isotopic values primarily reflect the protein fraction of the diet and tend to be dominated by animal protein inputs when present (Ambrose and Norr 1993), this discrepancy suggests that a non‐negligible contribution of plant‐derived proteins may be required to account for such low Δ^15^N_Glx–Phe_ values. Because the analyzed dentin section corresponds to a post‐weaning period, as indicated by the sequential bulk δ^15^N profile of the same tooth (Haas 2023), the low Δ^15^N_Glx–Phe_ value most likely reflects the composition of the post‐weaning childhood diet rather than breastfeeding. This interpretation is consistent with evidence for starch consumption at Khuzhir‐Nuge XIV based on dental calculus analyses (Clarke 2015).

## 5 Conclusion

This study confirms that δ^66^Zn is an effective proxy for refining understanding of dietary variation in prehistoric populations from Cis‐Baikal. Interestingly, fish zinc isotope ratios do not correlate with trophic levels but with dietary groups, with the low trophic level selective feeders surprisingly showing the lower δ^66^Zn values.

By integrating two different dietary proxies, δ^66^Zn with CSIA‐AA, this study provides direct evidence that trophic level (Δ^15^N_Glx–Phe_) and protein origin (δ^15^N_Phe_) do not fully resolve dietary variability when multiple aquatic resources are consumed. Fish and seals present overlapping δ^15^N_Phe_ values, but their Zn concentrations differ substantially—making δ^66^Zn uniquely suited to detecting seal exploitation. This allowed us to distinguish individuals regularly consuming seal from those who did not at Shamanka II. Phenylalanine δ^15^N values remain a very important parameter indicator of protein source. At Khuzhir‐Nuge XIV, elevated δ^15^N_Phe_ values support a dominant terrestrial protein input, even among individuals interpreted as consuming aquatic resources. In contrast, lower δ^15^N_Phe_ values at Shamanka II indicate a markedly greater contribution of aquatic resources relative to Khuzhir‐Nuge XIV.

In the specific context of Lake Baikal, δ^66^Zn appears to be a particularly practical proxy for identifying regular consumption of seals. Given the consistency between δ^66^Zn values and dietary interpretations established in the Baikal Archaeology Project, this proxy could be reliably extended to other contexts involving high‐trophic aquatic or terrestrial mammals—such as seals, orcas, large sharks, or potentially even mustelids.

## Author Contributions

**Gwen Le Bras:** writing – original draft, visualization, investigation, formal analysis, conceptualization, software, methodology, writing – review and editing, data curation, resources. **Erin Jessup:** validation, visualization, writing – review and editing, data curation, resources, methodology. **Christelle Lagane:** methodology, investigation, resources, writing – review and editing. **David Point:** methodology, validation, supervision, conceptualization, investigation, project administration, resources, writing – review and editing. **Rick J. Schulting:** validation, writing – review and editing, data curation, investigation, project administration, resources, supervision. **Victoria van der Haas:** methodology, investigation, resources, writing – review and editing. **Ian Scharlotta:** methodology, investigation, resources, writing – review and editing. **Stéphanie Mandrou:** methodology, investigation, resources, validation, writing – review and editing. **Olga I. Goriunova:** methodology, investigation, resources, writing – review and editing. **Vladimir I. Bazaliiskii:** methodology, investigation, resources, writing – review and editing. **Andrzej W. Weber:** supervision, conceptualization, validation, methodology, project administration, data curation, funding acquisition, investigation, resources, writing – review and editing. **Klervia Jaouen:** supervision, conceptualization, project administration, methodology, funding acquisition, writing – review and editing, data curation, investigation, resources, software, writing – original draft.

## Funding

This work was supported by the European Research Council (803676) and the Baikal Archaeological Project.

## Ethics Statement

This study is based exclusively on curated archeological human remains from Neolithic and Bronze Age contexts in the Cis‐Baikal region. The material derives primarily from excavations conducted since the late 20th century and is curated by academic institutions in the Irkutsk region, in collaboration with Irkutsk State University, within the framework of the Baikal Archaeology Project.

## Supporting information


**Figure S1:** Chromatogram of the AA‐mix standard solution measured by GC‐IRMS for mass 28 (N_2_). Each labeled peak represents an identified amino acid signal, plotted according to its retention time (in seconds) and scaled to its peak intensity (mV). Reference‐gas pulses are displayed before and after the sample signal. An axis break is applied to the *x*‐axis on this figure.
**Figure S2:** Analytical quality control plots for Zn and nitrogen CSIA‐AA measurements. (A) Mass‐dependent isotopic fractionation between δ^66^Zn and δ^68^Zn values. (B) Linear relationship between δ^15^N values of hydroxyproline and proline, reflecting their shared metabolic origin.
**Figure S3:** Linear relationships between δ^66^Zn and ^87^Sr/^86^Sr in humans and terrestrial fauna from the Cis‐Baikal region. A threshold value (dashed line) was derived from GFS individuals at Little Sea. Three models are presented: (1) terrestrial herbivores only, (2) omnivores (humans and canids), and (3) all terrestrial specimens combined. A significant correlation is observed only among omnivores (*p* = 7.3 × 10^−7^, *R* ^2^ = 0.343).
**Figure S4:** Relationship between δ^15^N_Phe_ and δ^66^Zn in Cis‐Baikal. The plot illustrates the relationship between δ^15^N_Phe_ and δ^66^Zn values for ecological categories (fish, seals, humans, terrestrial herbivores, and omnivores) in the Cis‐Baikal region. Individuals are represented as points, colored by microregion (e.g., Angara, SW Baikal, Little Sea) and shaped according to their ecological category. GFS/GF differentiation is also displayed with different color shade. The “Unknown location” category refers to a fish specimen documented only as originating from Lake Baikal, for which no more precise assignment was possible.
**Figure S5:** Bulk collagen δ^15^N and δ^13^C values of studied individuals from Little Sea and Southwest Baikal, colored by microregion and dietary category (GF and GFS).
**Figure S6:** Comparison of isotopic values between previously published data (by BAP research) and new measurements from this study. (A) Linear regression between δ^13^C values measured in collagen (‰). (B) Linear regression between δ^15^N values measured in collagen (‰). (C) Linear regression between ^87^Sr/^86^Sr values measured in teeth. Each data point represents a unique individual. The orange dot identifies individual *K14_1997.019*, which displays an unusually high ^87^Sr/^86^Sr value in the present dataset and was considered as outlier for a second linear model calculation. The dashed red regression line and associated statistics correspond to the dataset excluding this outlier.
**Table S1:** Summary of several linear regression models between δ^66^Zn and different trophic position estimators. Models were tested on subsets of archeological fish and human datasets. A negative slope consistently indicates decreasing δ^66^Zn values with increasing trophic level.
**Table S2:** List of sites and materials sampled in this study. Site locations can be seen on Figure 1. More information is given in Table S3. EN: Early Neolithic; EBA: Early Bronze Age.
**Figure S7:** Among the tested cluster configurations (*k* = 3 to 8), *k* = 5 was retained as the optimal solution, as it satisfied two complementary criteria: (i) the elbow in the within‐cluster sum of squares curve, indicating a diminishing gain in model fit beyond five clusters, and (ii) a relatively high average silhouette score, reflecting well‐separated and internally coherent clusters. Importantly, this k configuration also prevents over‐partitioning, as no cluster contains a single species, thereby supporting both its ecological relevance and statistical robustness.
**Figure S8:** Non‐metric multidimensional scaling used on fish species to identified dietary cluster. We used NMDS to reduce the dimensionality of the trophic matrix while preserving dissimilarities between species based on their dietary profiles. The final NMDS solution reached a stress value of 0.0666. K‐means cluster applied to the non‐metric scaling distribution of the fish species study in the present work with a number of clusters inferred at *k* = 5.

**Table S3:** Data.
**Table S4:** Fish Dietary Matrix.
**Table S5:** Ref. Materials.

## Data Availability

The data that support the findings of this study are available in the Supporting Information of this article.
