# Supplementary material for: Multi‐Isotopic Reconstruction of Prehistoric Diet and Trophic Organization in Cis‐Baikal Using Zinc and Amino Acid Nitrogen Isotopes
Source: Am J Biol Anthropol. 2026 Nov 9;191(3):e70396. doi: 10.1002/ajpa.70396 (PMC13746312; doi:10.1002/ajpa.70396)
Supplement: Supplementary file 1 [file AJPA-191-e70396-s001.docx]

# Multi-Isotopic Reconstruction of Prehistoric Diet and Trophic Organization in Cis-Baikal Using Zinc and Amino Acid Nitrogen Isotopes

Gwen Le Bras^1,^ Erin Jessup^2^, Christelle Lagane^1^, David Point^1^, Rick J Schulting^3^, Victoria van der Haas^2^, Ian Scharlotta^4^, Stéphanie Mandrou^1^, Olga I Goriunova^5^, Vladimir I Bazaliiskii^5^, Andrzej W Weber^2, 5^, Klervia Jaouen^1^

Affiliations:

^1^ Université de Toulouse, CNRS/IRD, Géosciences Environnement Toulouse, UMR 5563, Toulouse, France

^2^ Department of Anthropology, University of Alberta, Edmonton, AB, Canada

^3^ School of Archaeology, University of Oxford, UK

^4^ Texas State University, Center for Archaeological Studies, San Marcos, TX, USA

^5^ Research Centre “Baikal Region”, Irkutsk State University, Karl Marx Street 1, Irkutsk 664003, Russia

Corresponding authors: [gwenlebrassvt@gmail.com](mailto:gwenlebrassvt@gmail.com), [klervia.jaouen@cnrs.fr](mailto:klervia.jaouen@cnrs.fr)

**Keywords:** non-traditional stable isotopes; amino acids; Bronze Age; Neolithic; nitrogen isotopes; zinc; hunter-gatherers; freshwater ecosystems; dental enamel; trophic level; Baikal Archaeological Project

Figure S1 Chromatogram of the AA-mix standard solution measured by GC-IRMS for mass 28 (N_2_). Each labelled peak represents an identified amino acid signal, plotted according to its retention time (in seconds) and scaled to its peak intensity (mV). Reference-gas pulses are displayed before and after the sample signal. An axis break is applied to the x-axis on this figure.

Figure S2 Analytical quality control plots for Zn and nitrogen CSIA-AA measurements. (A) Mass-dependent isotopic fractionation between δ^66^Zn and δ^68^Zn values. (B) Linear relationship between δ^15^N values of hydroxyproline and proline, reflecting their shared metabolic origin.

Figure S3 Linear relationships between δ^66^Zn and ^87^Sr/^86^Sr in humans and terrestrial fauna from the Cis-Baikal region. A threshold value (dashed line) was derived from GFS individuals at Little Sea. Three models are presented: (1) terrestrial herbivores only, (2) omnivores (humans and canids), and (3) all terrestrial specimens combined. A significant correlation is observed only among omnivores (p= 7.3·10^-7^, R^2^ = 0.343).

Figure S4 Relationship between δ^15^N_Phe_ and δ^66^Zn in Cis-Baikal. The plot illustrates the relationship between δ^15^N_Phe_ and δ^66^Zn values for ecological categories (fish, seals, humans, terrestrial herbivores, and omnivores) in the Cis-Baikal region. Individuals are represented as points, colored by microregion (e.g., Angara, SW Baikal, Little Sea) and shaped according to their ecological category. GFS/GF differentiation is also displayed with different color shade. The “Unknown location” category refers to a fish specimen documented only as originating from Lake Baikal, for which no more precise assignment was possible.

Figure S5 Bulk collagen δ^15^N and δ^13^C values of studied individuals from Little Sea and Southwest Baikal, colored by microregion and dietary category (GF and GFS).

Figure S6 Comparison of isotopic values between previously published data (by BAP research) and new measurements from this study. (A) Linear regression between δ^13^C values measured in collagen (‰). (B) Linear regression between δ^15^N values measured in collagen (‰). (C) Linear regression between ^87^Sr/^86^Sr values measured in teeth. Each data point represents a unique individual. The orange dot identifies individual *K14_1997.019*, which displays an unusually high ^87^Sr/^86^Sr value in the present dataset and was considered as outlier for a second linear model calculation. The dashed red regression line and associated statistics correspond to the dataset excluding this outlier.

| Dataset | Trophic Proxy | Slope | Intercept | R^2^ | p-value (t-test) | n |
| --- | --- | --- | --- | --- | --- | --- |
| Fish only | TP_theo_ | –0.031 | 0.991 | 0.003 | 6.75e–1 | 59 |
| Fish only | TP_bulk_ | –0.180 | 1.446 | 0.062 | 1.61e–1 | 33 |
| Fish only | TP_AA_ | –0.677 | 3.939 | 0.563 | 3.00e–3 | 13 |
| Humans (Little Sea) + Terrestrial fauna | TP_AA_ | –0.332 | 1.713 | 0.847 | 1.00e–7 | 18 |
| Humans (SWB–GFS) + Fish + Seal | TP_AA_ | –0.403 | 2.377 | 0.164 | 2.60e–2 | 30 |
| Humans (Angara + SWB) + Fish + Seal | TP_AA_ | –0.341 | 2.115 | 0.156 | 1.60e–2 | 37 |

Table S1 Summary of several linear regression models between δ^66^Zn and different trophic position estimators. Models were tested on subsets of archaeological fish and human datasets. A negative slope consistently indicates decreasing δ^66^Zn values with increasing trophic level.

| Site | Micro-  region | Archaeological Period | Date | Geology | Species | No.  (Figure 1) |
| --- | --- | --- | --- | --- | --- | --- |
| Kitoi | Angara |  | Prehistoric | Limestones and dolomites | *Cervus elaphus* (n = 1)  *Sus scrofa* (n = 1) | 14 |
| Lokomotiv |  | Kitoi (EN) | 7,560–6,660 BP (Weber et al. 2021) |  | *Cervus elaphus* (n = 3)  *Homo sapiens* (n = 16)  *Marmota sibirica* (n = 1) | 8 |
| Gorelyi Les |  |  | Prehistoric |  | *Capreoleus pygargus* (n = 3)  *Cervus elaphus* (n = 4) | Gorelyi Les |
| Ostrov Listvenichnyi |  |  |  |  | *Capreolus pygargus* (n = 1) | 155 |
| Ust’-Belaia |  |  |  |  | *Equus sp.* (n = 2) | 19 |
| Ust’-Ida I |  |  |  |  | *Cervus elaphus* (n = 2) | 36 |
| Ust'-Khaita |  |  |  |  | *Acipenser sp.* (n = 2)  *Coregonus lavaretus* (n = 1) | Ust'-Khaita |
| Moty  (Lower Irkut) |  |  | Modern |  | *Abramis brama* (n = 1)  *Brachymystax lenok* (n = 1)  *Coregonus autumnalis* (n = 2)  *Coregonus lavaretus* (n = 1)  *Hucho taimen* (n = 3) | Moty |
| Moty-Novaia Shamanka  (Lower Irkut) |  |  | Modern |  | *Abramis brama* (n = 2)  *Brachymystax lenok* (n = 2)  *Coregonus autumnalis* (n = 1)  *Esox lucius* (n = 2)  *Gobio gobio* (n = 3)  *Leuciscus leuciscus* (n = 3)  *Lota lota* (n = 3)  *Perca fluviatilis* (n = 3)  *Thymallus arcticus* (n = 3) | 160 |
| Shamanka II (Group 2) | SW Baikal | Kitoi (EN) | 7,560–7,000 BP (Weber et al. 2025) |  | *Homo sapiens* (n = 15)  *Marmota sibirica* (n = 2)  *Ursus arctos* (n = 3) | 142 |
| Kultuk Bay |  |  | Modern | Metamorphic and igneous rocks | *Comephorus dybowskii* (n = 3)  *Cottocomephorus grewingkii* (n = 3)  *Esox lucius* (n = 2)  *Leocottus kesslerii* (n = 3)  *Perca fluviatilus* (n = 5)  *Rutilus rutilus* (n = 3) | Kultuk Bay |
| Bolshie Koty | W Baikal |  | Modern |  | *Leuciscus idus* (n = 2)  *Pusa sibirica* (n = 2)  *Thymallus baicalensis sp.* (n = 4) | Bolshie Koty |
| Lake Baikal | Lake Baikal |  | Modern |  | *Esox lucius* (n = 1)  *Leuciscus idus* (n = 1)  *Lota lota* (n = 1)  *Thymallus baicalensis* (n = 3) | Lake Baikal |
| Berloga | Little Sea |  | Prehistoric | Metamorphic and igneous rocks  Paleozoic sedimentary rocks  +  Quaternary alluvial deposits | *Canis sp.* (n = 2)  *Cervus elaphus* (n = 2)  Esox lucius (n = 1)  *Pusa sibirica* (n = 1) | Berloga |
| Ithyrkhei |  |  | Prehistoric |  | *Pusa sibirica* (n = 4) | Ithyrkhei |
| Kharansa I |  |  |  |  | *Cervus elaphus* (n = 2) | 130 |
| Khuzhir-Nuge XIV |  | Glazkovo (EBA) | 4970–3470 BP (Weber et al. 2021) |  | *Cervus elaphus* (n = 1)  *Homo sapiens* (n = 19) | 141 |
| Khuzhir-Nuge |  |  | Modern |  | *Coregonus baicalensis* (n = 2)  *Esox lucius* (n = 1) | Khuzhir-Nuge |
| Kurma XI |  |  | Prehistoric |  | *Cervus elaphus* (n = 3) | 138 |
| Little Sea |  |  | Modern |  | *Coregonus migratorius* (n = 1)  *Rutilus rutilus* (n = 2)  *Thymallus baicalensis brevipinnis* (n = 3) | Little Sea |
| Mys Uiuga |  |  | Prehistoric |  | *Canis sp.* (n = 4)  *Cervus elaphus* (n = 1)  *Sus scrofa* (n = 1) | 158 |
| Sagan Nuge III |  |  | Modern |  | *Acipenser sp.* (n = 1)  *Lota lota* (n = 1) | Sagan Nuge III |
| Sagan-Zaba II |  |  | Prehistoric  Modern |  | *Artiodactyla sp.* (n = 1)  *Bos sp.* (n = 4)  *Canis lupus familiaris* (n = 2)  *Capreolus pygargus* (n = 1)  *Cervus elaphus* (n = 2)  *Pusa sibirica* (n = 11)  *Ursus arctos* (n = 1) | Sagan-Zaba II |
| Shamanskii Mys |  |  | Prehistoric |  | *Pusa sibirica* (n = 5) | 129 |
| Tyshkine II and III |  |  | Prehistoric |  | *Pusa sibirica* (n = 8) | Tyshkine II and III |
| Ulan-Khada |  |  |  |  | *Canidae* (n = 4) | 115–119 |
| Upper Lena River | Upper Lena |  | Modern |  | *Hucho taimen* (n = 1) | Upper Lena River |
| Ust’-Kut | Upper Lena N |  | Modern | Paleozoic sedimentary rocks  +  Quaternary alluvial deposits | *Esox lucius* (n = 1) | Ust’-Kut |
| Obkhoi | Upper Lena S |  | Prehistoric |  | *Canis sp.* (n = 1) | 98 |
| Makrushino |  |  | Prehistoric |  | *Cervus elaphus* (n = 2) | 84 |
| Nikolskii Grot |  |  |  |  | *Sus scrofa* (n = 1) | 95 |

Table S2 List of sites and materials sampled in this study. Site locations can be seen on Figure 1. More information is given in Table S3. EN: Early Neolithic; EBA: Early Bronze Age.

# Supplementary Information 1. Materials and Methods

## SI 1.1. Archaeological Background

The Kitoi cultural pattern (Early Neolithic, 7560–6660 cal. BP) is geographically restricted to the Angara and Southwest Baikal microregions. In other areas, including the Little Sea and Upper Lena, the Late Mesolithic Khin tradition continued through the Early Neolithic without fully adopting the Kitoi economic, technological, and mortuary package (Weber 2020). The Kitoi pattern is characterized by distinctive material culture, large cemeteries with abundant use of ochre, substantial social differentiation, and sex-based associations of grave goods (Weber 1995; 2020; Weber et al. 2025). Technological innovations during this phase include the introduction of pottery and the bow and arrow.

The subsequent Early Bronze Age Glazkovo pattern (4970–3470 cal. BP) is widespread across Cis-Baikal and appears less socially differentiated than Kitoi groups. It displays technological continuity with earlier lithic industries alongside the use of copper objects such as fish hooks, needles, rings, and knives. Although broadly similar to earlier Neolithic toolkits, both lithic and organic, morphological differences are present.

Zooarchaeological research at Lokomotiv indicates that local fisheries were dominated by black grayling (*Thymallus baicalensis*, 80 %), lenok (*Brachymystax lenok*, 15 %), and taimen (*Hucho taimen*, 5 %) (Kozhov 1950; Weber et al. 2002; 2021). At Khuzhir-Nuge XIV, dietary group distinctions (GF vs GFS) have been interpreted as reflecting differential access to high-trophic-level aquatic resources such as seals, potentially linked to mobility, migration, or social organization (Weber and Bettinger 2010; Weber and Goriunova 2013).

## SI 1.2. Material

The complete dataset includes 230 samples: 50 humans and 180 faunal specimens (Table S2). Human tissues comprise dentine, dental collagen, and enamel from individuals excavated at Khuzhir-Nuge XIV (Little Sea), Lokomotiv (Angara), and Shamanka II (Southwest Baikal).

Faunal samples include teeth from cervids, bovids, equids, marmots, canids, and ursids, as well as seal teeth and fish bones. All terrestrial and seal specimens derive from Neolithic or Bronze Age contexts, whereas most fish samples are modern due to the limited preservation of archaeological fish remains in the region. Archaeological fish remains are poorly preserved and are particularly scarce in cemetery contexts, from which much of the archaeological faunal material analyzed in this study derives. Consequently, predominantly modern fish specimens were analyzed to characterize the δ^66^Zn variability of fish taxa relevant to prehistoric Cis-Baikal food webs. The modern specimens represent species known to have been present and available to human populations in the region during the Middle Holocene and are therefore considered suitable taxonomic proxies for their prehistoric counterparts. Greater caution is warranted for specimens from the Angara microregion, where the construction of three large hydroelectric dams during the 20^th^ century profoundly altered the riverine fishery and hydrological connectivity (Weber et al. 2011). Modern fish from this microregion may therefore be less representative of prehistoric ecological conditions than those from other areas of Cis-Baikal.

δ^66^Zn analyses were conducted on bone, enamel, and dentine from 193 individuals (198 measurements). δ^15^N compound-specific amino acid analyses were performed on 23 humans, 12 terrestrial mammals, 14 seals, and 13 fish. Dentine sections corresponding to enamel formation periods were selected for CSIA-AA to ensure temporal comparability between tissues.

Previously published bulk collagen isotope data (δ^13^C, δ^15^N) and strontium isotope ratios (^87^Sr/^86^Sr) from earlier BAP studies were incorporated into the comparative dataset (see Table S2 for references).

## SI 1.3. Methods

### SI 1.3.1. Collagen Extraction and Bulk Carbon and Nitrogen Isotope Analysis

Collagen was extracted from bone and dentin samples using a protocol based on Longin (1971) and Brown, Nelson, Vogel, & Southon (1988) and adapted after Fuller et al. (2014). Samples were demineralized in 0.5 M HCl at room temperature until complete decalcification. When necessary, an additional brief NaOH bath was applied to samples exhibiting visible soil contamination or discoloration to remove humic acids; this step was otherwise omitted. Gelatinization was conducted at 70 °C in weakly acidic solution (pH~3) for 24 to 48 hours. The resulting solution was filtered. The final solubilized product was lyophilized and weighed. Samples were analyzed by EA-IRMS for bulk carbon and nitrogen isotope ratios at Iso-Analytical Ltd. (UK). Measurements were calibrated against international reference materials traceable to VPDB (carbon) and AIR (nitrogen). In-house and international standards were also monitored (IA-R068, IA-R038, IA-R069, and IAEA-C7).

### SI 1.3.2. Detail for Zn and Sr Preparation and Analytical Procedures

For all tissues, the outermost surface was abraded using a Dremel® diamond burr to eliminate potential contamination from soil contact. Tools were cleaned with Milli-Q water between samples. All glassware and plasticware were soaked in 1 M HCl for 24 hours, rinsed with Milli-Q water, and soaked again in Milli-Q water for an additional 24 hours. Tubes and pipette tips underwent similar acid-cleaning procedures. All preparation was conducted in a metal-free, overpressured clean laboratory using ultrapure acids (upa).

The international standard SRM 987 yielded a mean ^87^Sr/^86^Sr value of 0.710271 ± 0.000001 (n = 12); isotope ratios were corrected relative to the accepted value of 0.71024 ± 0.00004.

### SI 1.3.3. CSIA-AA Procedures

SI 1.3.3.1. **Derivatization**

The collagen hydrolysis and amino acid derivatization procedures followed a modified version of Corr et al. (2007) and Honch et al. (2012), as fully described in Ma et al. (2021). Between 1 and 5 mg of purified collagen were transferred into pre-pyrolyzed glass vials, mixed with 0.5 mL of 6 M HCl, sealed, and heated at 110 °C for 20 h to release free amino acids. Following hydrolysis, samples were evaporated under a nitrogen flow at 60 °C and re-dissolved in 0.1 M HCl prior to derivatization. Derivatization into N-acetyl isopropyl esters (NAIPs) proceeded in two steps: first, esterification of carboxyl groups using a 4:1 2-propanol:acetyl chloride solution; second, acetylation of amino groups using a 1:2:5 acetic anhydride:trimethylamine:acetone mixture.

SI 1.3.3.2. **GC-IRMS Measurements**

GC-IRMS analyses were performed at Géosciences Environnement Toulouse using a Thermo Fisher MAT 253 mass spectrometer coupled via a ConFlo IV combustion interface to a Trace 1310 gas chromatograph equipped with a GC IsoLink II. Amino acids were separated on a custom VF-23 ms column (60 m × 0.32 mm, film thickness 0.5 µm) using helium as carrier gas at a constant flow rate of 1.5 mL/min. The GC oven was held at 70 °C for 0.5 min, ramped to 130 °C, then to 255 °C, and held at the final temperature until a total run time of 37 min. Samples were injected in splitless mode with an injection volume of 2 µL.

An internal standard of L-Norleucine was added to each sample, prior to derivatization. Additionally, international reference materials USGS65 and USGS74 were included in each batch and regularly analyzed. Each sequence also included in-house standards provided by the Memorial University of St John’s, Cana-da—SW (sperm whale) Canadian Whale Standard, FBTR (C3 cow) and Bgel (C4 cow)—whose CSIA results are reported in data Table S4. Each sample was injected three times consecutively, with ethyl-acetate injected between triplications. This approach minimized potential contamination and drift through-out the sequence.

### SI 1.3.4. Fish Diet Clustering

SI 1.3.4.1. **Choosing the Number of Clusters**

Figure S7 Among the tested cluster configurations (k = 3 to 8), k = 5 was retained as the optimal solution, as it satisfied two complementary criteria: (i) the elbow in the within-cluster sum of squares curve, indicating a diminishing gain in model fit beyond five clusters, and (ii) a relatively high average silhouette score, reflecting well-separated and internally coherent clusters. Importantly, this k configuration also prevents over-partitioning, as no cluster contains a single species, thereby supporting both its ecological relevance and statistical robustness.

SI 1.3.4.2. **K-means Clustering with k = 5**

Figure S8 Non-metric multidimensional scaling used on fish species to identified dietary cluster. We used NMDS to reduce the dimensionality of the trophic matrix while preserving dissimilarities between species based on their dietary profiles. The final NMDS solution reached a stress value of 0.0666. K-means cluster applied to the non-metric scaling distribution of the fish species study in the present work with a number of clusters inferred at k = 5.
